# Supplementary material for: Inequality in economic shock exposures across the global firm-level supply network
Source: Nat Commun. 2024 Apr 18;15:3348. doi: 10.1038/s41467-024-46126-w (PMC11026497; doi:10.1038/s41467-024-46126-w)
Supplement: Supplementary file 1 — Supplementary Information [file 41467_2024_46126_MOESM1_ESM.pdf]

Supplementary information for "Inequality in economic shock exposures across the global firm-level supply network"

Abhijit Chakraborty,[1,*] Tobias Reisch,[1,2,*] Christian Diem,[1,3]
Pablo Astudillo-Estévez,[4,5,1] and Stefan Thurner[2,1,6,†]
[1]*Complexity Science Hub Vienna, A-1080 Vienna, Austria*
[2]*Section for Science of Complex Systems, CeMSIIS, Medical University of Vienna, A-1090 Vienna, Austria*
[3]*Institute for Finance, Banking and Insurance, Vienna University of Economics and Business, A-1020 Vienna, Austria*
[4]*Universidad San Francisco de Quito & School of Economics, Quito 170902, Ecuador*
[5]*Institute for New Economic Thinking, University of Oxford, Oxford, OX1 3QY, UK*
[6]*Santa Fe Institute, Santa Fe, NM 85701, USA*

SUPPLEMENTARY INFORMATION

SI Text 1: DebtRank

The DebtRank R_i of a firm in a supply network [1] describes the overall reduction in economic activity subsequent to the default of an initial firm i and the cascade of defaults caused by it. Here, we provide a brief review of the underlying cascading mechanism and show how to calculate the indicator R_i as used in [1].

Let us consider a directed and weighted network with nodes $i = 1, 2, ..., N$. A link from node j to i has a weight $w_{ji} \in [0, 1]$ that represents a relative dependency of i to j. At any time-step t, the nodes are characterised by their state $S_i(t)$ and the amount of financial distress $h_i(t) \in [0, 1]$. The state of a node $S_i(t) \in \{A, D, I\}$ can be "Active", "Distressed" or "Inactive" at time t. We start with the following initial configurations:

$$h_i(0) = \begin{cases} 1, & \text{if } i \in \mathcal{M} \\ 0, & \text{otherwise} \end{cases} \tag{1}$$

and

$$S_i(0) = \begin{cases} D, & \text{if } i \in \mathcal{M} \\ A, & \text{otherwise} \end{cases} \tag{2}$$

where $\mathcal{M}$ is an initial set of "Distressed" nodes, which can be a single node as well. We update the amount of distress as

$$h_i(t) = \min\left[1, \quad h_i(t-1) + \sum_{j:S_j(t-1)=D} w_{ji} h_j(t-1)\right] \quad , \tag{3}$$

where the summation is taken over all the neighbours of i having state $S_j(t-1) = D$. We also update the state of each node simultaneously as follows

$$S_i(t) = \begin{cases} D, & \text{if } h_i > 0 \text{ and } S_i(t-1) = A \\ I, & \text{if } S_i(t-1) = D \\ S_i(t-1), & \text{otherwise} \end{cases} \tag{4}$$

Note that at the next time step, a node in state D becomes I, which does not propagate any distress to others afterwards. This helps to exclude, in the case of cycles, an infinite number of repercussions in shock propagation. However, an I node continues to receive distress from its distressed neighbours without affecting others. The propagation terminates after a finite number of time steps T. In the following we set $\mathcal{M} = \{i\}$ to just consider the default

* Equal contribution
† Corresponding author: stefan.thurner@meduniwien.ac.at

of one node, the generalization to a set of nodes is straightforward. We use the matrix D with element $D_{ij} = h_j(T)$ denoting the distress firm j receives if firm i defaults (the loss in economic production firm j experiences if firm i defaults). The column j contains the shock firm j is exposed to. Row i contains the shock firm i causes to all other firms j when it defaults. The total amount of distress, i.e. the total loss of production, in the system due to the initially distressed node i is measured as its DebtRank

$$R_i = \frac{\sum_j D_{ij} q_j}{\sum_j q_j} \quad , \tag{5}$$

where q_i is the size of the nodes i. We include the effect of the initial set of distressed nodes. The quantities discussed in the main text are based on different aggregations of D_{ij}.

Since our global supply chain network does not have link-weights and node size information, we have assigned each edge equal weight $w_{ji} = 1/k_i^{in}$ and associate the node-size with it's degree $q_i = k_i$, where k_i and k_i^{in} represent degree and in-degree of the i-th node, respectively. We choose degree k_i as size proxy, to be consistent and self-contained in the supply network dataset. However, one could also use other size proxies such as value added, turnover or employees.

SI Text 2: Robustness checks of the shock propagation mechanism

The results presented in the main text were obtained with a shock spreading mechanism calibrated from first principles, following [1]. Here, we present an alternative formulation and compare it to empirical impacts found after supplier defaults.

We start by reviewing the literature on firm-level output reductions after a supplier failure. *Barrot & Sauvagnat* [2] study output changes for firms in the USA after they or their suppliers were affected by a natural disaster. They find an average output reduction of -3.1% four quarters after the firm's supplier was affected by a natural disaster. This number can rise up to -5.0% if a 'specific' supplier is affected. *Carvalho et al.* [3] study output reductions of firms in Japan after the Great East Japan Earthquake of 2011. They find that firms' output is reduced by -3.8% if the firm's supplier was in the disaster affected area. The value rises to -4.2% if the supplier was in area flooded by the Tsunami caused by the earthquake. Finally, *Boehm et al.* [4] studied cross-country spread of economic shocks. They show that close affiliates of Japanese corporations in the USA experienced drops in output after the Great East Japan Earthquake directly proportional to the output drops of their Japanese suppliers —"approximately Leontief".

We can compare these values to the average output reduction after a supplier default, $\langle \Delta h_i \rangle$, expected in our model. From Eq. (3) we can easily see that after a single supplier defaults we expect an output reduction of

$$\langle \Delta h_i \rangle = \langle w_{ij} \rangle = \langle 1/k_j^{in} \rangle \quad . \tag{6}$$

For the network used in our study we find $\langle \Delta h_i \rangle = 0.543$, i.e. the expected output reduction after a supplier fails is -54.3%.

This is significantly larger than the empirical values reported in the literature. To correct for this issue, we adapt Eq. (3) by introducing the shock-damping factor σ_{ij},

$$h_i(t) = \min \left[1,\ h_i(t-1) + \sum_{j:S_j(t-1)=D} (\sigma_{ji} w_{ji} h_j(t-1)) \right] \quad . \tag{7}$$

We follow [?] and use σ to model that, in the short term, it is not easily possible to establish links to firms in far-away countries, and that firms with larger market shares are harder to replace. We define the replacement factor as the suppliers output relative to the national market size at time t,

$$\sigma_{ji}(t) = \min \left[\frac{k_j(0)}{\sum_{l \in sector_j \ \wedge \ l \in country_i} k_l(0)(1 - h_l(t))}, 1 \right] \quad . \tag{8}$$

This results in an average output reduction after supplier default of $\langle \Delta h_i \rangle = \langle \sigma_{ij} w_{ij} \rangle = 0.045$, an output reduction of -4.5%, a value of similar size as in the literature discussed above.

Figure S1a shows the results of the robustness check using the replacement factor for E^{cd}_{down}. The characteristic features of the *country-country exposure* discussed in the main text, high values along the diagonal, blocks corresponding to continents, and horizontal lines of high exposure created by rich European, Asian and North American countries. In Fig. S1 we plot the matrix elements of both E^{cd} with and without damping factor against each other. We find that they are highly correlated with a Pearson correlation of $r = 0.998, p < 10^{-10}$. In Fig. S1c we compare the total exposure, E^{d}_{down}, to GDP per capita. Also here we can reproduce the finding from the main text, a negative correlation between the two quantities (Pearson $r = 0.44, p < 10^{-5}$).

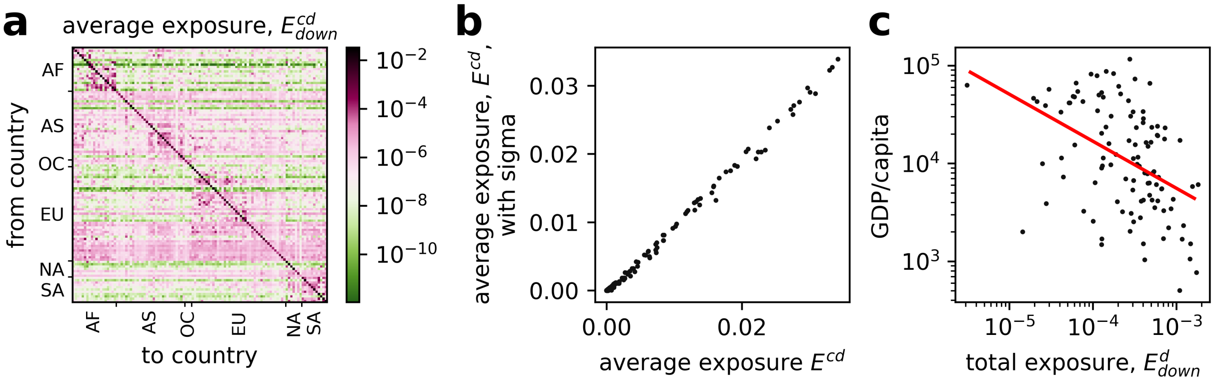

SI Fig. S1. Testing the robustness to a shock damping factor σ, simulating replacement within country and sector. (a) Average exposure E^{cd}, as shown in main text Fig. 2. (b) Scatter plot of the original matrix elements of E^{cd} and matrix elements E^{cd} including the damping factor. The exposures both ex- and including the damping factor are highly correlated. (c) Comparison of total exposure E^d and GDP per capita. The two values are negatively correlated. The cascade including the damping factor is highly correlated with E^{cd} and reproduces all the main results presented in the main text.

SI Text 3: Expected loss $\tilde{E}^{cd}$ with heterogeneous probabilities of default

In the main text we present the results of the *average exposure*, E^{cd} of country d conditional that a random firm in country c is removed. Here we discuss the effect of assigning every firm i a certain probability of failing, i.e. its probability of default p_i, with which E^{cd} is actually realized. We call this new quantity *expected loss* $\tilde{E}^{cd}$ [5],

$$\tilde{E}^{cd} = \sum_{i \in \mathcal{C}^c} \frac{p_i E_i^d}{|\mathcal{C}^c|} \quad . \tag{9}$$

If the probabilities of default are constant for all firms, $p_i \equiv p$, we obtain the result simply by multiplying it with E^{cd},

$$\tilde{E}^{cd} = \sum_{i \in \mathcal{C}^c} \frac{p E_i^d}{|\mathcal{C}^c|} = p \sum_{i \in \mathcal{C}^c} \frac{E_i^d}{|\mathcal{C}^c|} = p E^{cd} \quad . \tag{10}$$

However, in practice not all firms have equal probability of default. In the following we test the robustness of our results by calculating $\tilde{E}^{cd}$ with heterogeneous p_i.

First, we sample p_i for every firm from a Beta distribution, $p(x) \propto x^{\alpha-1}(1-x)^{\beta-1}$, with $\mu(p_i) = 0.01$ and $\sigma(p_i) = 0.04$, corresponding to $\alpha = 0.05$ and $\beta = 4.95$, and shown as orange curve in Fig. S2. Figure S3a shows $\tilde{E}^{cd}$, with values below 10^{-12} cropped. We find that the block structure described in the main text persists, just the overall size of the losses is two orders of magnitude smaller. This is because it is the expected exposure and not the average over exposures which is conditional on the firm's default. In Fig S3b we plot $\tilde{E}^{cd}$ against E^{cd}. The results are highly correlated ($r = 0.83, p < 10^{-15}$). Figure S3c shows $\tilde{E}^d = \sum_c \tilde{E}^{cd}$, analogous to Fig. 3b in the main text. We find a significant negative correlation of $r = -0.30, p < 0.002$.

Second, we sample a random probability of default for every sector from a uniform distribution, $p^{ind} \in [0.005, 0.01]$. Then we sample p_i for every firm from Beta distribution with $\mu(p_i) = p_i^{ind}$ and $\sigma(p_i) = 0.04$. Figure S4a shows the resulting $\tilde{E}^{cd}$, with values below 10^{-12} cropped. We find that the block structure described in the main text persists, just the overall size of the losses is two orders of magnitude smaller for the same reason as above. In Fig S4b we plot $\tilde{E}^{cd}$ against E^{cd}. The results are still highly correlated ($r = 0.74, p < 10^{-15}$). Figure S4c shows $\tilde{E}^d = \sum_c \tilde{E}^{cd}$, analogous to Fig. 3b in the main text. We find a significant negative correlation of $r = -0.42, p < 10^{-5}$.

We find that the main results of our paper, the block-diagonal structure of E^{cd} and the negative correlation of E^d with GDP per capita, are still valid when heterogeneous probabilities of default are taken into account in the *expected loss* $\tilde{E}^{cd}$ and $\tilde{E}^d$, respectively.

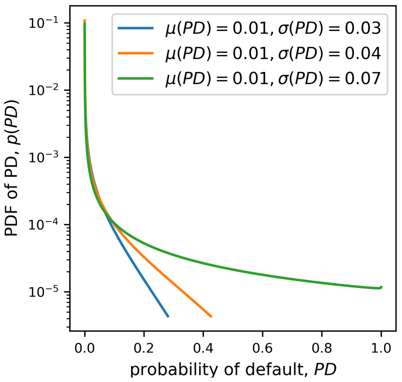

SI Fig. S2. Beta distributions with mean $\mu = 0.01$ and standard deviations $\sigma = 0.03$ (blue), $\sigma = 0.04$ (orange), and $\sigma = 0.07$ (green). The orange curve is implemented the simulations presented in this SI Text.

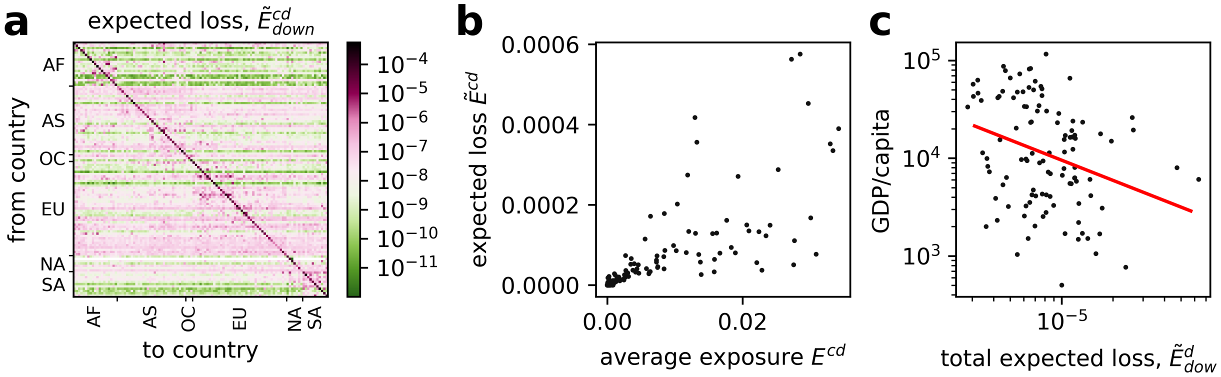

SI Fig. S3. Testing the robustness of our results to heterogeneous probabilities of default sampled from a Beta distribution with $\mu = 0.01$ and $\sigma = 0.04$. (a) Expected loss $\tilde{E}^{cd}$, showing the expected loss country d suffers from defaults in country c. Note that values below 10^{-12} are cropped. (b) Scatter plot of the original matrix elements of E^{cd}, without probabilities of default, and matrix elements $\tilde{E}^{cd}$, including probabilities of default. (c) Comparison of total expected loss $\tilde{E}^{d}$ and GDP per capita. The two values are negatively correlated The results for expected loss are highly correlated with E^{cd} and reproduces all the main results presented in the main text.

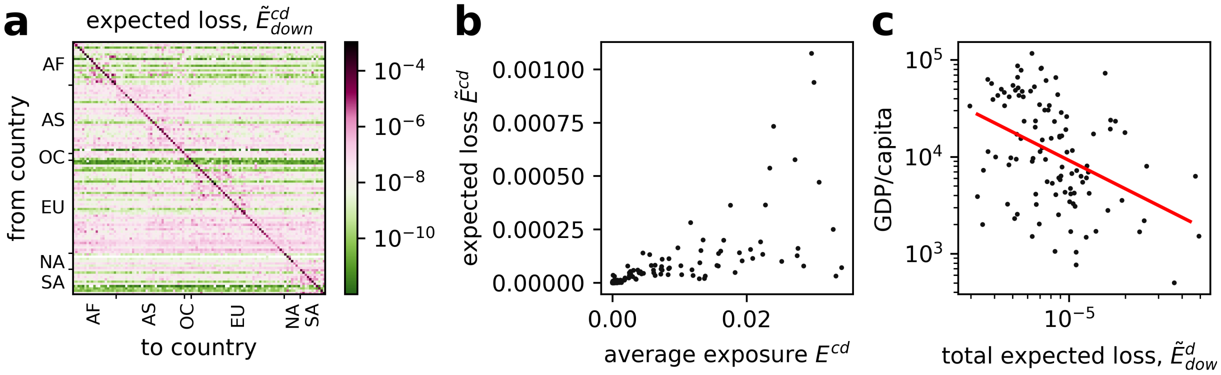

SI Fig. S4. Testing the robustness of our results to heterogeneous probabilities of default sampled from a Beta distribution with $\mu \in [0.005, 0.01]$ different for every industry, sampled from a uniform distribution and $\sigma = 0.04$. (a) Expected loss $\tilde{E}^{cd}$, showing the expected loss country d suffers from defaults in country c. Note that values below 10^{-12} are cropped. (b) Scatter plot of the original matrix elements of E^{cd}, without probabilities of default, and matrix elements $\tilde{E}^{cd}$, including probabilities of default. (c) Comparison of total expected loss $\tilde{E}^{d}$ and GDP per capita. The two values are negatively correlated The results for expected loss are highly correlated with E^{cd} and reproduce all the main results presented in the main text.

SI Text 4: Toy example of a cascade of production interruptions

In SI Fig. S5 we show the cascade subsequent to the default of firm 2. Compared to the default of firm 1, country C is hit harder and country B is not affected at all.

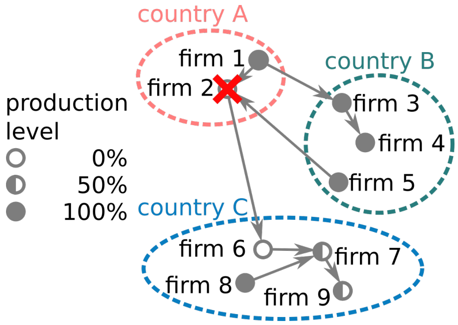

SI Fig. S5. Visualization of a cascade of production reductions, in a toy economy of three nations and nine firms, subsequent to the default of firm 2, marked by a red cross. The filling of the nodes (pie chart) indicates their remaining production level after the shock propagation. The production of a firm is reduced proportional to the reduction in trade at the supplier divided by the number of suppliers the firm has.

In the main text we mention that, if firm 5 would supply firm 1 rather than firm 2, the resulting cascade would be much larger. In SI Fig. S6a we show the cascade firm 5 causes in the original network; compared to Fig. S6b, where we show the cascade firm 5 would cause in the rewired network. In the rewired example, the cascade can also spread to country B, which was previously unaffected, and country C is affected more strongly because firm 2 is more susceptible to shocks from firm 1, now that firm 1 is its only supplier. The cascade after the rewiring is significantly larger and some firms which were previously not affected now fully collapse.

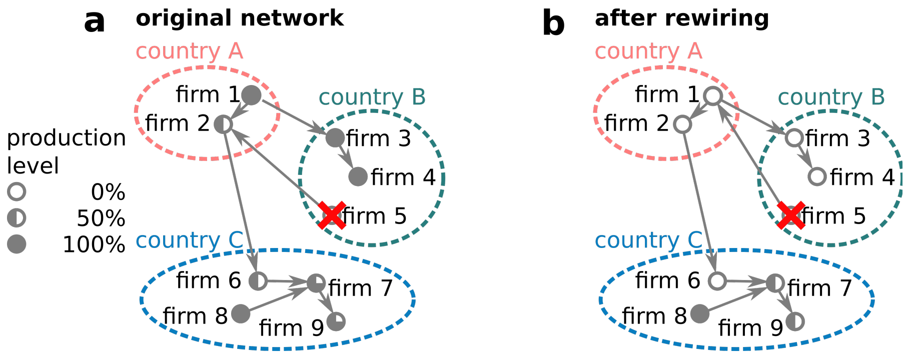

SI Fig. S6. Details of the production network structure strongly affect shock spreading. (a) Shock spreading after the failure of firm 5 in the original network as shown in main text Fig. 1. (b) Cascade caused by firm 5 if it links to firm 1 instead of firm 2. The cascade after the rewiring is significantly larger and some firms which were previously not affected now fully collapse.

SI Text 5: Detailed exposure matrix

In the main text we show that the expected downstream exposure between countries, E^{cd}_{down}, is clearly structured by geographic regions. Exposures are highest within countries, then within regions and continents. In SI Fig. S7 we show a large version of the same plot, such that we can label the individual countries. The numbers correspond to the index column in SI Tab. S1.

In Africa (AF) we find two blocks corresponding to north African and sub-Sahara countries. Most of their high exposures are within their region, but it is also instructive to consider the columns of the matrix, showing that, e.g. some north African countries are strongly exposured to Asia and Europe, but not to the Americas.

In Asia (AS) we have three regions, East Asia and Pacific, Middle East and South Asia, but observe only two prominent blocks, suggesting that the regional classification does not fully represent economic regions. Several Asian countries expose countries around the world to medium exposure, highlighted by horizontal white lines.

Oceania and Australia (OC) does not show up as a prominent block, but is rather connected to South Asia. Further, as an industrialized region it creates exposure to most other countries in the world.

The European (EU) countries ares split into four groups, Central and Eastern Europe (CEE), Northern Europe (NE), Southern Europe (SE), and Western Europe (WE). CEE and NE countries form relatively well separated blocks, while SE and WE highly expose all countries. This strongly suggests that SE and WE countries are integrated into the world market more strongly than NE and CEE countries.

In North America (NA) there is no clear block structure visible. However, for Canada (index 97), Mexico (98) and the USA (99) the horizontal white lines show that they highly expose most countries around the globe. The magenta vertical line for the USA between row 100 and 110 suggests that the USA is also highly exposed to disruptions in Southern America.

South America (SA) is visible as a very prominent, dark magenta block. This implies that the economies in SA are tightly interwoven and expose each other strongly.

TABLE S1: Geographical grouping of countries into continents, regions and income groups.

index	country name	continent	region	income group
0	Algeria	Africa	Middle East and North Africa	low
1	Egypt	Africa	Middle East and North Africa	low
2	Libya	Africa	Middle East and North Africa	middle
3	Morocco	Africa	Middle East and North Africa	low
4	Tunisia	Africa	Middle East and North Africa	low
5	Angola	Africa	Sub-Saharan Africa	low
6	Botswana	Africa	Sub-Saharan Africa	middle
7	Côte d'Ivoire	Africa	Sub-Saharan Africa	low
8	Ghana	Africa	Sub-Saharan Africa	low
9	Kenya	Africa	Sub-Saharan Africa	low
10	Mozambique	Africa	Sub-Saharan Africa	low
11	Mauritius	Africa	Sub-Saharan Africa	middle
12	Namibia	Africa	Sub-Saharan Africa	low
13	Nigeria	Africa	Sub-Saharan Africa	low
14	Tanzania, United Republic of	Africa	Sub-Saharan Africa	low
15	Uganda	Africa	Sub-Saharan Africa	low
16	South Africa	Africa	Sub-Saharan Africa	middle
17	Zambia	Africa	Sub-Saharan Africa	low
18	Zimbabwe	Africa	Sub-Saharan Africa	low
19	China	Asia	East Asia and Pacific	middle
20	Hong Kong	Asia	East Asia and Pacific	high
21	Indonesia	Asia	East Asia and Pacific	low
22	Japan	Asia	East Asia and Pacific	high
23	Cambodia	Asia	East Asia and Pacific	low
24	Korea, Republic of	Asia	East Asia and Pacific	high
25	Macao	Asia	East Asia and Pacific	high
26	Mongolia	Asia	East Asia and Pacific	low
27	Malaysia	Asia	East Asia and Pacific	middle
28	Philippines	Asia	East Asia and Pacific	low
29	Singapore	Asia	East Asia and Pacific	high
30	Thailand	Asia	East Asia and Pacific	middle
31	Taiwan	Asia	East Asia and Pacific	high
32	Viet Nam	Asia	East Asia and Pacific	low
33	United Arab Emirates	Asia	Middle East and North Africa	high
34	Bahrain	Asia	Middle East and North Africa	high
35	Iran, Islamic Republic of	Asia	Middle East and North Africa	low
36	Israel	Asia	Middle East and North Africa	high
37	Jordan	Asia	Middle East and North Africa	low
38	Kuwait	Asia	Middle East and North Africa	high
39	Lebanon	Asia	Middle East and North Africa	middle
40	Oman	Asia	Middle East and North Africa	middle
41	Qatar	Asia	Middle East and North Africa	high
42	Saudi Arabia	Asia	Middle East and North Africa	high
43	Turkey	Asia	Middle East and North Africa	middle
44	Bangladesh	Asia	South Asia	low
45	India	Asia	South Asia	low
46	Sri Lanka	Asia	South Asia	low

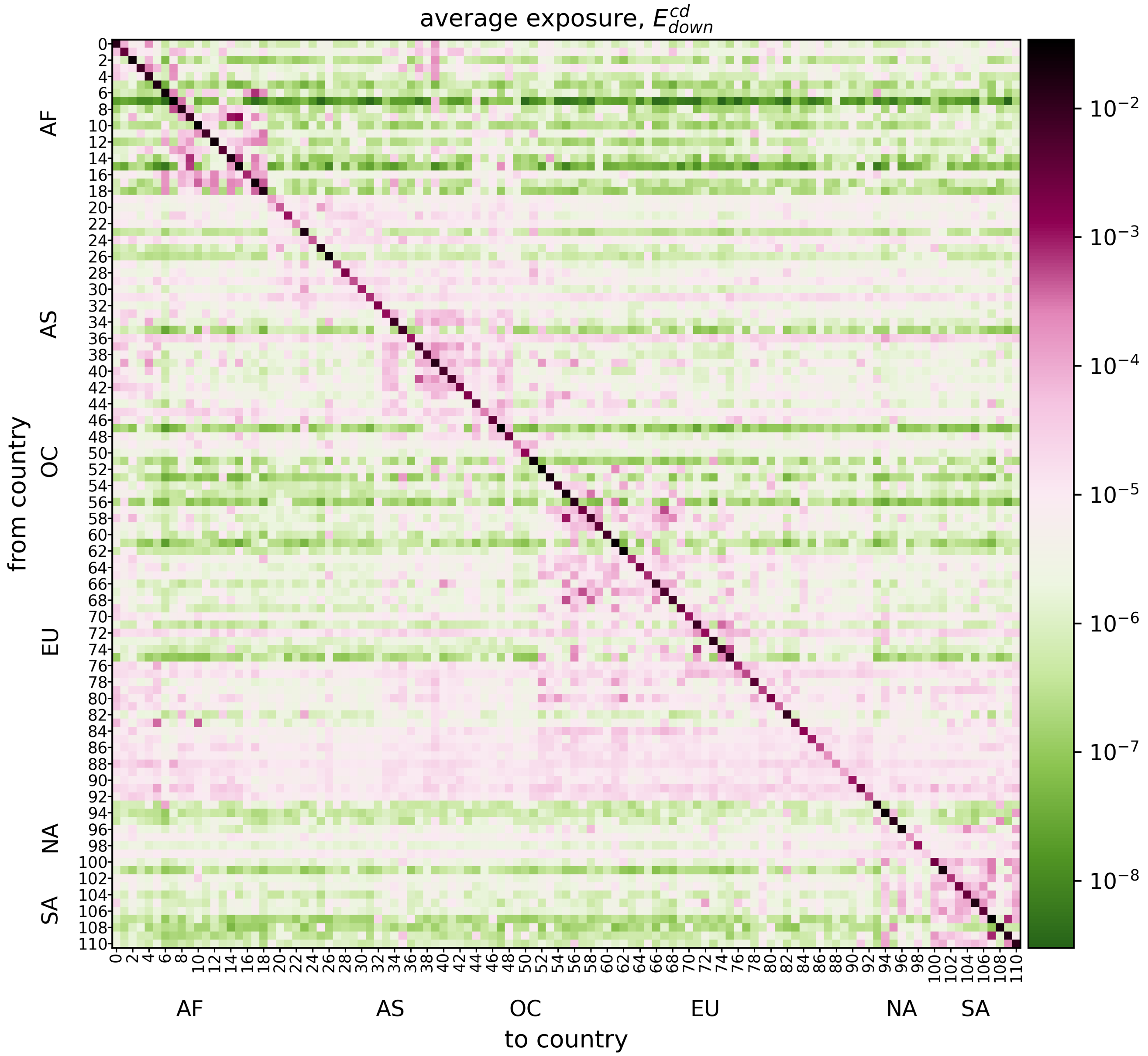

SI Fig. S7. The network structure of international downstream exposure. Average exposure E^{cd}_{down} sorted by continent and region on a logarithmic scale. The continents are sorted as follows: Africa (AF), Asia (AS), Oceania and Australia (OC), European (EU), North America (NA), and South America (SA). The numbering corresponds to the index in SI Tab. S1, where the countries' name and region are listed.

TABLE S1: Continuation:

index	country name	continent	region	income group
47	Nepal	Asia	South Asia	low
48	Pakistan	Asia	South Asia	low
49	Australia	Australia and Oceania	East Asia and Pacific	high
50	New Zealand	Australia and Oceania	East Asia and Pacific	high
51	Papua New Guinea	Australia and Oceania	East Asia and Pacific	low
52	Armenia	Europe	Central and Eastern Europe	low
53	Azerbaijan	Europe	Central and Eastern Europe	low

TABLE S1: Continuation:

index	country name	continent	region	income group
54	Bulgaria	Europe	Central and Eastern Europe	middle
55	Bosnia and Herzegovina	Europe	Central and Eastern Europe	low
56	Belarus	Europe	Central and Eastern Europe	middle
57	Czechia	Europe	Central and Eastern Europe	high
58	Croatia	Europe	Central and Eastern Europe	middle
59	Hungary	Europe	Central and Eastern Europe	middle
60	Kazakhstan	Europe	Central and Eastern Europe	middle
61	Moldova, Republic of	Europe	Central and Eastern Europe	low
62	North Macedonia	Europe	Central and Eastern Europe	middle
63	Poland	Europe	Central and Eastern Europe	middle
64	Romania	Europe	Central and Eastern Europe	middle
65	Russian Federation	Europe	Central and Eastern Europe	middle
66	Serbia	Europe	Central and Eastern Europe	middle
67	Slovakia	Europe	Central and Eastern Europe	middle
68	Slovenia	Europe	Central and Eastern Europe	high
69	Ukraine	Europe	Central and Eastern Europe	low
70	Denmark	Europe	Northern Europe	high
71	Estonia	Europe	Northern Europe	middle
72	Finland	Europe	Northern Europe	high
73	Iceland	Europe	Northern Europe	high
74	Lithuania	Europe	Northern Europe	middle
75	Latvia	Europe	Northern Europe	middle
76	Norway	Europe	Northern Europe	high
77	Sweden	Europe	Northern Europe	high
78	Cyprus	Europe	Southern Europe	high
79	Spain	Europe	Southern Europe	high
80	Greece	Europe	Southern Europe	middle
81	Italy	Europe	Southern Europe	high
82	Malta	Europe	Southern Europe	high
83	Portugal	Europe	Southern Europe	high
84	Austria	Europe	Western Europe	high
85	Belgium	Europe	Western Europe	high
86	Switzerland	Europe	Western Europe	high
87	Germany	Europe	Western Europe	high
88	France	Europe	Western Europe	high
89	United Kingdom	Europe	Western Europe	high
90	Ireland	Europe	Western Europe	high
91	Luxembourg	Europe	Western Europe	high
92	Netherlands	Europe	Western Europe	high
93	Bahamas	North America	Latin America and Caribbean	high
94	Dominican Republic	North America	Latin America and Caribbean	middle
95	Jamaica	North America	Latin America and Caribbean	low
96	Panama	North America	Latin America and Caribbean	middle
97	Canada	North America	North America	high
98	Mexico	North America	North America	middle
99	United States	North America	North America	high
100	Argentina	South America	Latin America and Caribbean	middle
101	Bolivia, Plurinational State of	South America	Latin America and Caribbean	low
102	Brazil	South America	Latin America and Caribbean	middle
103	Chile	South America	Latin America and Caribbean	middle
104	Colombia	South America	Latin America and Caribbean	middle
105	Ecuador	South America	Latin America and Caribbean	middle
106	Peru	South America	Latin America and Caribbean	middle
107	Paraguay	South America	Latin America and Caribbean	low
108	Trinidad and Tobago	South America	Latin America and Caribbean	middle
109	Uruguay	South America	Latin America and Caribbean	middle
110	Venezuela, Bolivarian Republic of	South America	Latin America and Caribbean	low

SI Text 6: Correlation of direct links and country country exposure

Direct trade links represent first order (i.e. direct) exposures. Consequently, the *exposed value* in country c subsequent to the default of a firm in country d, V^{cd}, increases as function of the average number of links firms in country c have to country d. We quantify the direct influence of one country on another by the average number of out-links of a firm in country c to the firms in country d, $\bar{k}^{cd} = A^{cd}/N^c$. In Fig. S8 we plot V^{cd} on the y-axis and $\bar{k}^{cd}$ on the x-axis. We find that V^{cd} spans five orders of magnitude. Although we observe a high correlation between $\bar{k}^{cd}$ and V^{cd} (Pearson's $r = 0.93$ $p < 10^{-15}$), we find large variations in V^{cd}, for a given level of average outlinks. The red arrow in SI Fig. S8 highlights that this variation can be more than two orders of magnitude for a given value of average out-degree. This variation can be attributed to higher order exposures that are heavily influenced by the network topology. For a fully connected network –equivalent to an aggregated version of the network– V^{cd} and $\bar{k}^{cd}$ are perfectly correlated.

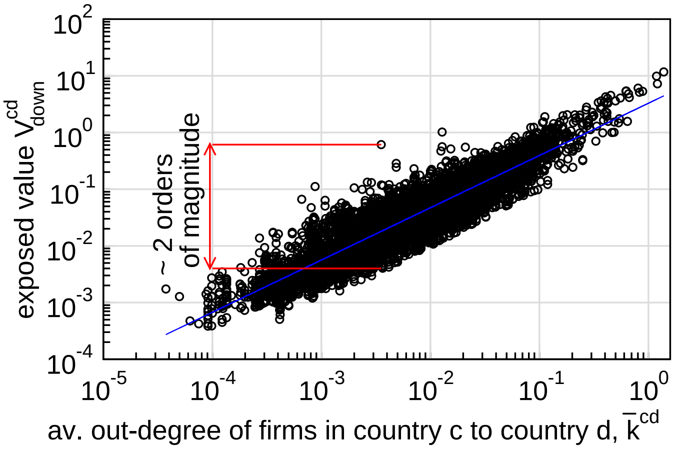

SI Fig. S8. Exposed economic value V^{cd}_{down} is plotted against the average number of out-links of firms in country c to country d, $\bar{k}^{cd}$. The blue line represents the regression line: $V^{cd}_{down} \sim \bar{k}^{cd\ 0.92}$ estimated by a log-log least square fit. Although V^{cd}_{down} correlates strongly with $\bar{k}^{cd}$, for a given level of connectedness, $\bar{k}^{cd}$, the exposed value, V^{cd}_{down}, ranges over two orders of magnitude.

SI Text 7: Gravity models

Gravity models are well established in international economics to explain bilateral trade flows [6, 7]. They are named after and inspired by the law of gravity, where the gravitational attraction between two point masses is proportional to the product of their masses divided by the square of their distance. For trade, the countries' export and import volumes take the role of 'mass' and distance is typically approximated by the distance between the countries' center of mass or capitals.

The standard formulation for 'Gravity models' for trade [6, 7] generalizes on the physical law of gravitation by allowing the exponents of the countries' sizes and the distance to deviate from one and two, respectively,

$$X_{cd} = G\frac{Y_c^{\beta_1} Y_d^{\beta_2}}{D_{cd}^{\beta_3}} \quad . \tag{11}$$

Here X_{cd} denotes the trade or exchange of some quantity X between countries or regions c and d, Y_c denotes the size of c (in terms of the given context), and D_{cd} denotes the geographical distance between c and d. The coefficients G, β_1, β_2, and β_3 are the parameters to be fitted.

Following the standard procedure, we transform to log-variables and apply an ordinary least squares (OLS) regression.

$$\log(X_{cd}) = \log(G) + \beta_1 \log(Y_c) + \beta_2 \log(Y_d) + \beta_3 \log(D_{cd}) + \epsilon$$

Note that we write the input to the OLS with a positive sign for the last term, hence we expect β_3 to be negative in the regression table.

TABLE S2. Regression table for the gravity models fitted to the number of links between countries A_{cd} and the exposure between countries, E_{cd}. For the gravity model of the number or links, A^{cd}, we set Y_c and Y_d to the total out- and indegree, k_c^{out} and k_d^{in}, respectively. For the gravity model of the average exposure, E^{cd}, we set Y_c and Y_d to the total outgoing and ingoing exposure, E_c^{from} and E_d^{to}, respectively.

	$\log(A^{cd})$	$\log(E^{cd})$
$\log(D_{cd})$	-0.53***	-0.66***
	(0.01)	(0.01)
$\log(k_d^{in})$	0.65***	
	(0.01)	
$\log(k_c^{out})$	0.61***	
	(0.01)	
$\log(E_{to}^{d})$		-0.77***
		(0.01)
$\log(E_{from}^{c})$		0.04***
		(0.01)
$\log(G)$	0.32	-6.43***
	(0.20)	(0.22)
R-squared Adj.	0.68	0.44
N	5513	11772

The first column in Tab. S2 shows the results for the number of links between countries c and d, A^{cd}. For D_{cd} we use the shortest distance between the capitals of country c and d, and for Y_c and Y_d from Eq. (11) we use country c's *total outdegree*, $k_c^{out} = \sum_d A^{cd}$, and country d's *total indegree*, $k_d^{in} = \sum_c A^{cd}$. The model explains 68% of the variance and has, as expected, positive exponents for the in- and outdegree and a negative exponent for the countries' distance.

The second column in Tab. S2 shows the results for the expected downstream exposure, E^{cd}_{down} between countries c and d. Again, we use the shortest distance between the capitals of country c and d for D_{cd}. For Y_c and Y_d from Eq. (11) we use country c's *total outgoing exposure*, $E^c_{out} = \sum_d E^{cd}$ and country d's *total exposure*, $E^d_{in} = \sum_c E^{cd}$. The model explains 44% of the variance and, as in the model for A^{cd}, the exponent of the distance is negative. However, the exponent for the receiving exposure of country d is highly negative and the exponent of the outgoing exposure of c country is very small but positive. This shows that the countries which experience a lot of distress, do so not mainly due to the countries creating large exposure, but by being exposed evenly to many countries. This discrepancy between the 'typical' gravity model for the number of links and the 'anomalous' model for risk exposure highlights the main finding of our paper that risk spreads differently from regular trade flows. In Fig. S9 we compare the prediction of the gravity model with the emperically found expected exposure, E^{cd}_{down}.

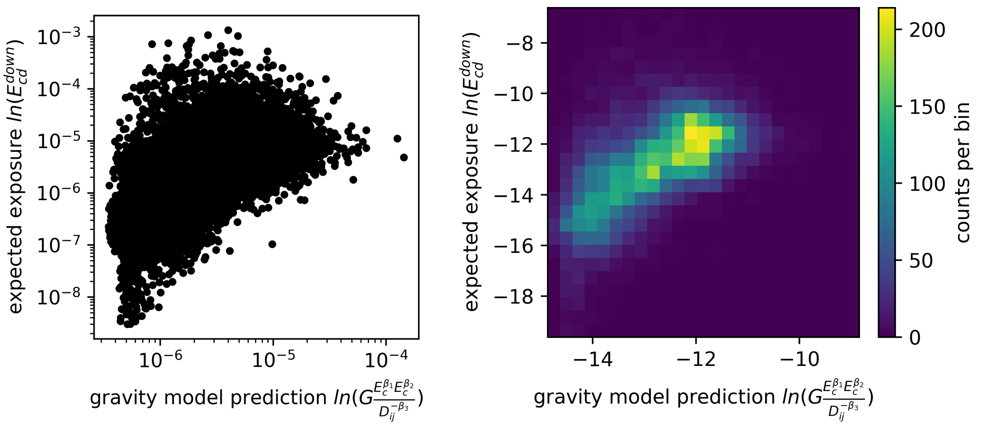

SI Fig. S9. Exposure predicted by the gravity model versus exposure calculated on the actual network. (a) Scatter plot of the Country-Country exposure E_{cd} as predicted by the gravity model in Tab. S2 and the corresponding matrix elements presented in Fig. 2. (b) Same data as in panel (a), but as heatmap. The values are well aligned along the diagonal.

SI Text 8: Multilinear regression model for GDP per capita

We perform the following multi linear regression model for GDP per capita and report the results in SI Tab. S3. We denote the error term with ϵ.

log(GDP/capita) $\sim$ log(Total downstream imported distress) + log(Exports) + log(Imports) + log(GDP) + Intercept + ϵ

The model explains 52% of variance (adjusted $R^2 = 0.52$) and E^d_{down} remains significant in the presence of the controls.

TABLE S3. Multi linear regression table for GDP/capita.

	log(GDPpc)
log(Ed)	-0.61**
	(0.24)
log(exports)	1.64***
	(0.29)
log(imports)	-0.79**
	(0.35)
log(GDP)	-0.64***
	(0.16)
Intercept	0.44
	(1.48)
R-squared	0.52
R-squared Adj.	0.50
N-Observations	105

Standard errors in parentheses.
* p<0.1, ** p<0.05, ***p<0.01

SI Text 9: GDP growth vs. total exposure E^d

Intuitively we expect higher gains from higher risks. For the lack of a better indicator, in SI Fig. S10 we compare total exposure E^d_{down} with average annual growth of GDP per capita. Whereas international trade is generally associated with higher GDP growth [8, 9], we find no significant correlation between E^d_{down} and the average GDP per capita growth rate over 20 years, between 1998 and 2018 (Pearson $r = 0.15, p = 0.12$), see SI Fig. S10. We test our results for robustness by calculating the correlation with 5- and 10-year growth, but find no correlations (10-year growth: Pearson $r = 0.11, p = 0.26$; 5-year growth: Pearson $r = -0.03, p = 0.73$). Note that we are limited by data availability and can only compare exposures on the 2017 network with historical growth rates.

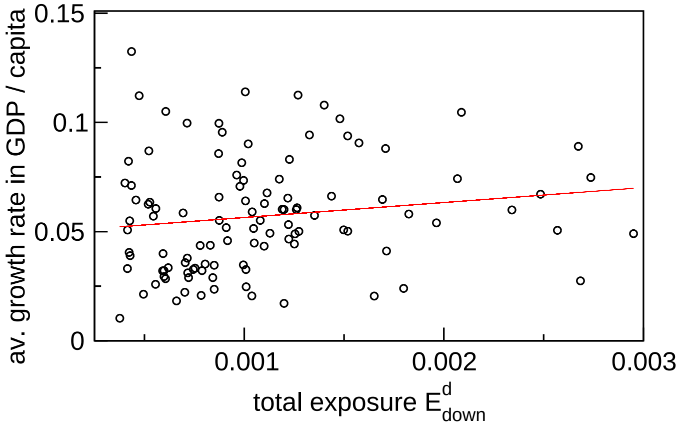

SI Fig. S10. Average growth in GDP per capita over 20 years is plotted against total exposure E^d_{down}. The red line represents the best linear fit to the data of form $y = 0.05 + 6.85x$. There is no significant linear relationship between the two variables.

The total exposure to downstream economic shocks doesn't correlate significantly with the growth of GDP per capita. However, other measures linked more directly international trade might still show the 'risk premium' we hypothesize. One such measure is Foreign direct investment (FDI), which is generally thought of as a good indicator for the upsides of "offshoring" and international trade, connected to, e.g., higher knowledge transfer and growth [10]. We can easily imagine that countries which are exposed to direct and indirect downstream shocks also receive more FDI from the countries they are exposed to. In SI Fig. S11 we compare the average net inward flows of FDI (in current US$, source:`https://unctadstat.unctad.org/datacentre/dataviewer/US.FdiFlowsStock`) between 1998 and 2018 and total downstream exposure E^d_{down}. The two quantities are not significantly correlated, Pearson's $r = -0.13$, $p = 0.21$. We test our results for robustness by calculateing the correlation with 5- and 10-year avearages, but find no significant correlations (5-year average: Pearson $r = -0.14$, $p = 0.18$; 10-year average: Pearson $r = -0.12$, $p = 0.25$).

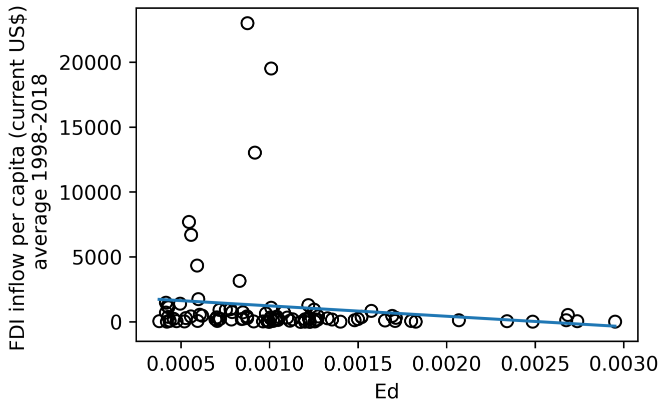

SI Fig. S11. Average net FDI inflow over 20 years (in current US$) is plotted against total exposure E^d_{down}. The red line represents the best linear fit to the data of form $y = 2028.5 - 801,500x$. There is no significant linear relationship between the two variables.

SI Text 10: Upstream exposures

In the main text, we have studied the downstream propagation of shocks, reflecting the impact of a supplier default. We can also study upstream cascades to show the impact of a customer's default resulting in propagating demand reductions. SI Figure S12 shows the variation of upstream exposed economic value V_{up}^{cd} with average in-degree of firms in country c from the firms in country d, $\bar{k}^{dc}$. Here also, we observe that these two quantities are strongly correlated ($r = 0.88, p < 10^{-15}$), but retain a lot of variance, indicated by a red arrow which shows a variation of two orders of magnitude for a given value of $\bar{k}^{dc}$.

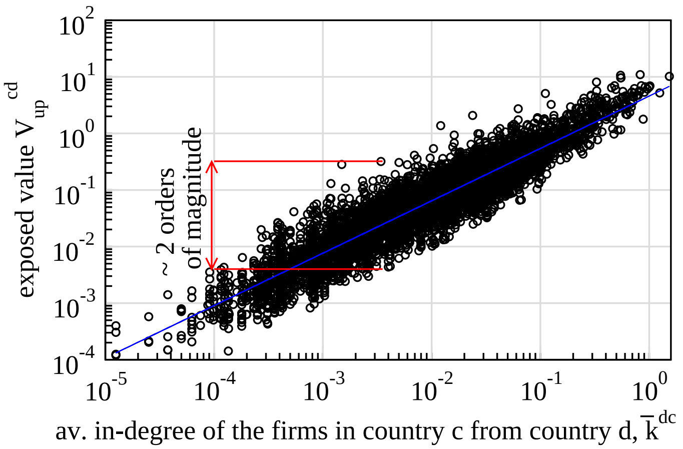

SI Fig. S12. The Exposed economic value for upstream V_{up}^{cd} is plotted with average in-degree of firms in country c from firms in country d, $\bar{k}^{dc}$. The blue line represents the best power-law fit to the data of form: $V_{up}^{cd} \sim (\bar{k}^{dc})^{0.93}$.

We explore the structure of the upstream country-country exposure matrix E_{up}^{cd} in SI Fig. S13. Contrary to intuition, it is not simply the transpose of its downstream counterpart. It shows similar structures, such as high values within countries and similar clusters of geographic regions. Confirming expectation, some countries that created a lot of downstream exposure, such the block of northern, southern and western European countries, now receive upstream distress when shocks pass buyer-supplier relations the opposite way. However, some countries, such as several Middle Eastern countries, very prominently create a lot of upstream exposure that was not visible in the downstream cascade, see SI Fig. S13.

We show the average upstream distress exposure matrix between firms in low, middle and high income countries in SI Fig S14a. It shows that firms in low and middle income countries affect each other more than any other pair of groups. High income countries on average experience little and create little exposure.

We plot the total upstream exposure E_{up}^{d} with GDP per capita of the countries in SI Fig S14b. It shows that these two quantities are negatively correlated ($r = -0.16, p < 0.04$), reflecting the fact that countries with lower GDP per capita receive more upstream imported distress, however with a weaker effect and lower significance.

We investigate the concentration of exposure to upstream cascades with the Lorentz curve in SI Fig S15. Again, E_{up}^{d} is concentrated more strongly than GDP with a Gini coefficient of 0.81, compared to a Gini coefficient of 0.59 for GDP.

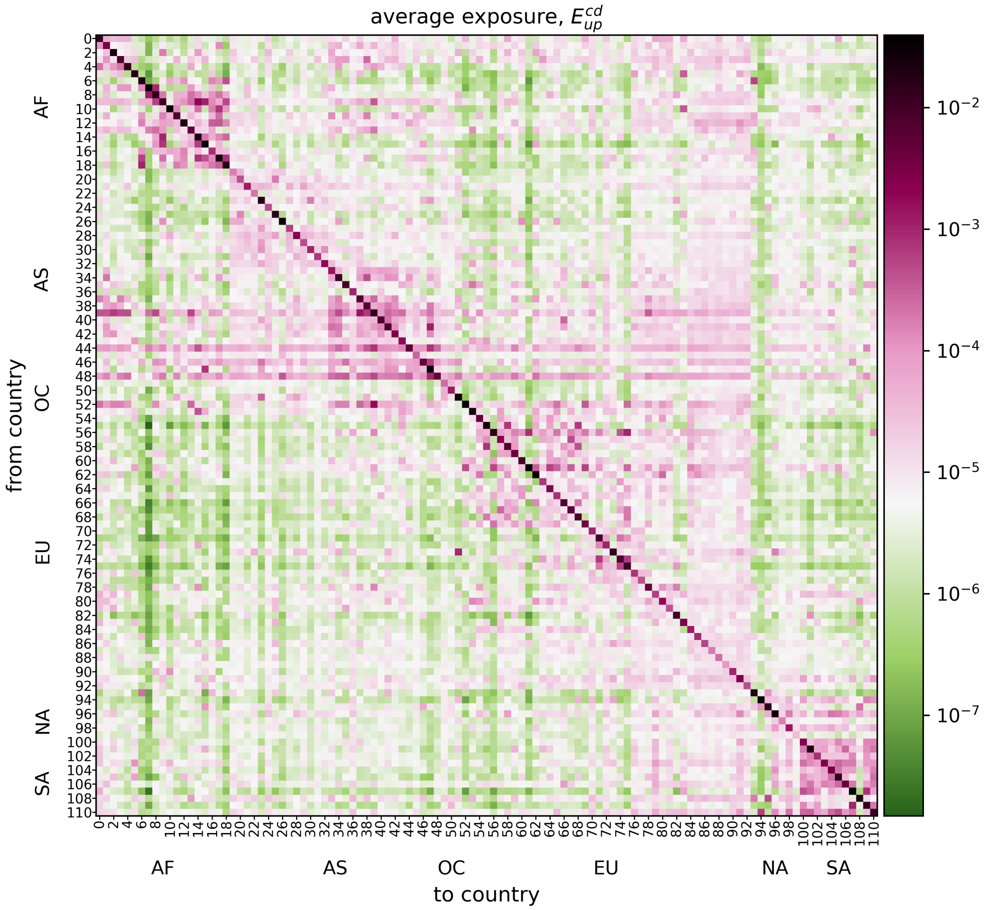

SI Fig. S13. The bilateral upstream distress (log-scale) for all pairs of countries is shown as a heat map. The countries are grouped according to their regional classification. The country names and their regional classification can be found in SI Tab. S1. The matrix resembles a transposed E_{down}^{cd} in terms of the block structure and the exposures of western Europe, but also highlights some differences. For instance, several Asian countries create high upstream exposures, such as Lebanon (index 19), Bangladesh (44), Sri Lanka (46) and Pakistan (48).

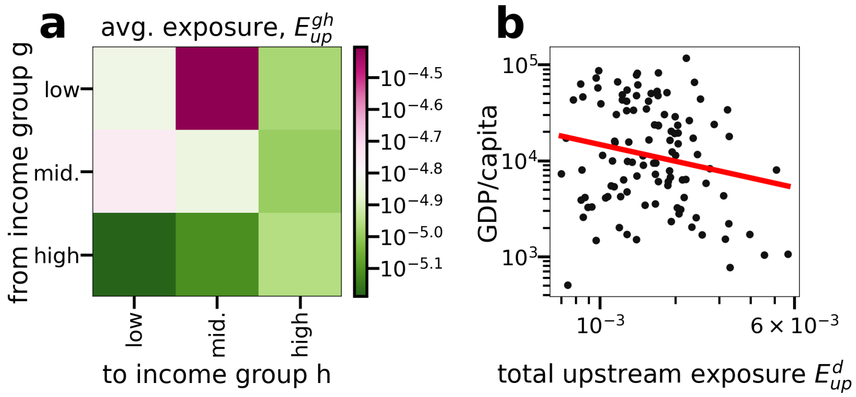

SI Fig. S14. Country income and upstream exposure. (a) Income group assortativity of average upstream exposure. Distress (log-scale) between firms separated into low-, middle- and high-income groups based on their country's GDP per capita. Large exposures are mostly between low and middle income countries, high income countries do not experience much exposure. SI Table S1 lists the income group for each country. (b) GDP per capita plotted against total upstream exposure E_{up}^{d}. A significant negative correlation of $r = -0.20, p < 0.04$ highlights that higher exposure is connected to lower income per capita. The red line represents the log-log ordinary least squares regression fit to the data of form $y \sim x^{-0.58}$.

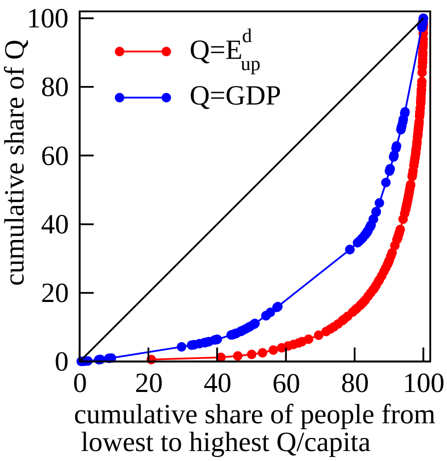

SI Fig. S15. Lorenz curves for total upstream imported distress and GDP of all the countries. The red (blue) line shows the proportion of risk (GDP) that is assumed by the lowest exposed (poorest) x% of people globally. For perfectly equally distributed exposure (wealth) the curve coincides with the diagonal, inequality is higher if the area between the curve and the diagonal increases. Exposure to economic shock is distributed more unequally than GDP. The area between the diagonal and the curve is proportional to the Gini index. We find Gini coefficients of 0.81 and 0.59 for E_{up}^{d} and GDP, respectively.

SI Text 11: Normalizing E^{cd}_{down} with country degree

To avoid a potential size effect of large countries creating more exposure, we normalize the *Country-Country Exposure*, E^{cd}, with the number of firms in the origin country c, $E^{cd} = \sum_{i\in\mathcal{C}^c} E^d_i/|\mathcal{C}^c|$. In practice, however, large countries can sustain (on average) larger companies. This is observable in the super-linear relation between a country's number of firms, $N^{firms}_c = |\mathcal{C}^c|$, and its total degree, $k_c = \sum_{i\in\mathcal{C}^c} k_i$. An OLS fit of the logarithmic variables confirms that $k_c \propto (N^{firms}_c)^{1.13}$, see also SI Fig. S16.

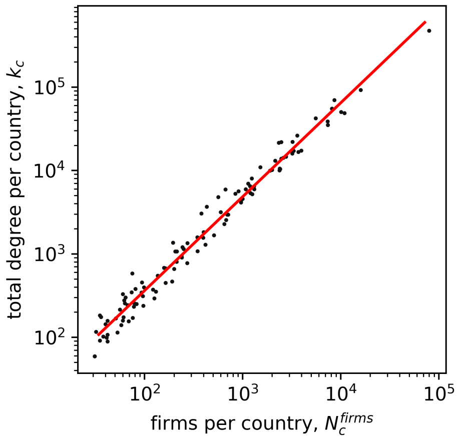

SI Fig. S16. Scaling relation of number of firms in a country, N^{firms}_c, and country total degree, k_c. The two variables are highly correlated with a Pearson coefficient of $r = 0.99$, $p < 10^{-10}$ and a Spearman coefficient of $\rho = 0.99$, $p < 10^{-10}$. The red line represents a best fit of the form $k_c \propto (N^{firms}_c)^{1.13}$.

By introducing the *degree normalized Country-Country Exposure*, $E^{*,cd} = \sum_{i\in\mathcal{C}^c} E^d_i/k_c = E^{cd}|\mathcal{C}^c|/k_c$, we normalize in a way that is robust to the fact that a country's average degree can be size dependent. In SI Fig. S17a we show that $E^{*,cd}_{down}$ shows similar features as E^{cd}_{down} (see Fig. 2a), the largest values are along the diagonal, a block-diagonal structure highlights that exposures are higher within economic regions, and bright horizontal lines indicate that some countries create exposures to almost all other countries. The features are fainter than in main text Fig. 2a, but clearly visible. In SI Fig S17b we plot the *total degree normalized exposure*, $E^{*,d}_{down}$, versus GDP per capita showing an anti-correlation (Pearson $r = -0.51$, $p < 10^{-7}$), which is only marginally lower than for E^d_{down} (shown in the main text).

The robustness of our results to a degree normalization in $E^{*,cd}$ suggests that they are not driven by the size of the exposing country, but because their firms are embedded differently into the global supply network.

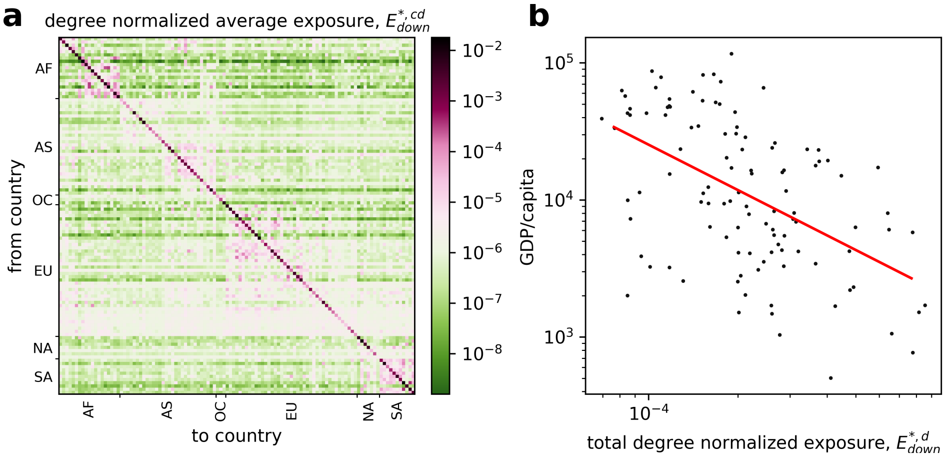

SI Fig. S17. Degree normalized Country-Country Exposure in the global supply network. (a) Degree normalized Country-Country Exposure, $E^{*,cd}$, showing the average loss country d suffers from defaults in country c. Note that values below 10^{-12} are cropped. (b) Comparison of total expected loss $\tilde{E}^{*,d}$ and GDP per capita. The two values are negatively correlated (Pearson $r = -0.51$, $p < 10^{-7}$). The red line represents the log-log ordinary least squares regression fit to the data of form $y \sim x^{-1.10}$. The results for $E^{*,cd}$ and $E^{*,d}_{down}$ qualitatively reproduce the main results presented in the main text.

SI Text 12: Representativeness of the global supply network data – robustness to missing nodes, links, and link weights

Our study is subject to several potential biases introduced by the quality of the data employed. First, the dataset we use in this study comprises 230,970 firms, out of a possible number that is in likely in the order of 300 millions [11]. This could potentially induce a sample bias, as the firms represented in the dataset tend to be large, internationally acting companies. A second potential sample bias is the relatively low density of the international supply network. In SI Tab S4 we report an average degree of $\bar{k} = 5.72$, which is significantly lower than the average degrees of $\bar{k} \approx 60 - 100$ reported for national production networks reconstructed from high-quality value added tax (VAT) data, such as Hungary, Japan, Belgium, the Dominican Republic or Ecuador (see Fig. 2 in [12]). Third, another potential bias is that we're not considering link weights, which are known to be highly skewed in supply networks [12].

In this SI Text we systematically investigate the robustness of our results to the data quality issues described above. The basic approach we choose is to first simulate the shock spreading algorithm described in the Methods section and SI Text 1 on a network which we consider as *ground truth*. Second, we either (i) remove nodes, (ii) remove links, or (iii) add weights, and re-run the shock spreading algorithm. Finally, we compare the results of the cascades on both the *ground truth* and *modified* network to assess biases introduced by each data quality issue. To this end, we calculate the correlation coefficient of the individual nodes' DebtRank, R_i, see SI Text 1 eq. (5), calculated on the *ground truth*, R_i^t, and *modified*, R_i^m, networks.

Removal of firms from the supply network

We start by investigating consequences of the fact that the firms contained in our dataset represent only a fraction of all global firms. The dataset we consider as *ground truth* in this exercise is the global supply network obtained from S&P's Capital IQ platform, as presented in the main text.

First, we randomly sample 10% of the nodes with uniform probability and calculate their induced subgraph. In SI Fig. S18a we compare the DebtRank of the 23,097 sampled firms on the induced subgraph, $R_i^{0.1,unif}$, with their DebtRank in the full network, R_i^t. The dynamics on the sampled network is highly representative of the dynamics on the full network, with a Pearson correlation coefficient of $r(R_i^t, R_i^{0.1,unif}) = 0.89$ ($p \approx 0.0$) and a Spearman correlation of $\rho(R_i^t, R_i^{0.1,unif}) = 0.71$ ($p \approx 0.0$). Note that $R_i^{0.1,unif}$ is typically larger than R_i^t, as can be seen by the coefficient of the linear fit: $R_i^{0.1,unif} = 4.7 R_i^t + 2 \cdot 10^{-5} + \epsilon$. An examination of the scaling relation $R_i^{0.1,unif} \propto (R_i^t)^{0.89}$ (red broken line) reveals that high values of systemic risk seem to be underestimated relatively to low values. The omission of nodes with uniform probability seems to weaken the observed effect.

The firms represented in the S&P Capital IQ database are typically large, internationally active companies, and not at all sampled with uniform probability. In a second exercise, we account for this by removing the smallest nodes and considering the induced subgraph of the 10% largest firms. SI Figure S18b correlates the node's DebtRank on the full network, R_i^t, with their DebtRank calculated on the modified network, $R_i^{0.1,largest}$. For this sampling strategy, the dynamics on the induced subgraph are highly representative of the dynamics on the full network, with a Pearson

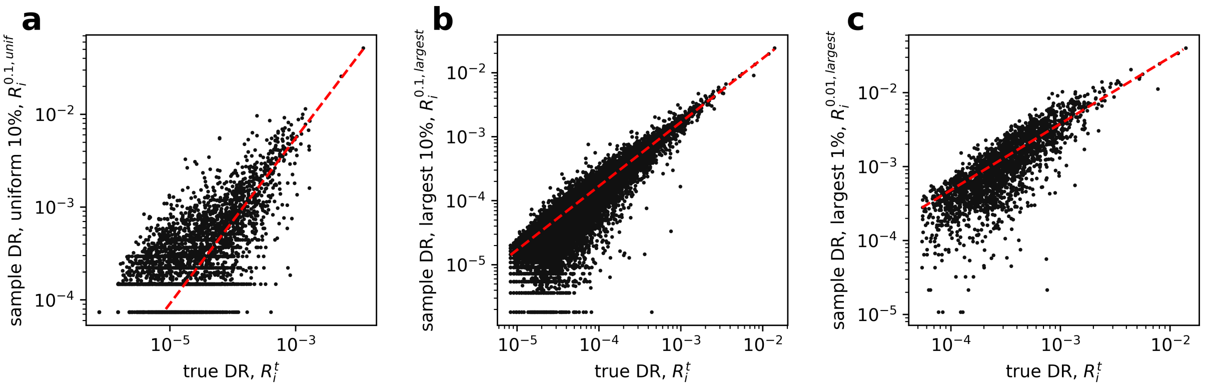

SI Fig. S18. Effect of missing firms in the global supply network data. (a) DebtRank calculated on the full global supply network, R_i^t, and on the induced subgraph of 10% of the nodes, sampled with uniform probability, $R_i^{0.1,unif}$. The red broken line shows a best fit $R_i^{0.1,unif} \propto (R_i^t)^{0.89}$. (b) DebtRank calculated on the full global supply network, R_i^t, and on the induced subgraph of the 10% largest nodes, $R_i^{0.1,largest}$. The red broken line shows a best fit $R_i^{0.1,largest} \propto (R_i^t)^{1.00}$. (c)DebtRank calculated on the full global supply network, R_i^t, and on the induced subgraph of the 1% largest nodes, $R_i^{0.01,largest}$. The red broken line shows a best fit $R_i^{0.01,largest} \propto (R_i^t)^{0.90}$. For all three sampling strategies, DebtRank calculated for the sample is highly representative of a node's DebtRank on the full network.

correlation coefficient of $r(R_i^t, R_i^{0.1,largest}) = 0.98$ ($p \approx 0.0$) and a Spearman correlation of $\rho(R_i^t, R_i^{0.1,largest}) = 0.91$ ($p \approx 0.0$). Note that also $R_i^{0.1,largest}$ is typically larger than R_i^t, as can be seen by the coefficients of the linear fit: $R_i^{0.1,largest} = 1.7R_i^t - 2.6 \cdot 10^{-6} + \epsilon$. An examination of the scaling relation $R_i^{0.1,largest} \propto (R_i^t)^{1.00}$ (red broken line) reveals that removing the 90% smallest nodes doesn't introduce a non-linear bias. For a better interpretation of these results, we reproduce the main results of the paper. SI Figure S19a shows E_{down}^{cd} for the induced subgraph of the 10% largest nodes. The results remain qualitatively the same, we still observe the highest values along the diagonal, a block structure, and horizontal lines. We confirm the higher exposure within the blocks using a Mann-Whitney U test. The test rejects the Null hypothesis that the average exposure within and outside of a country's continent is the same at a significance level of $p < 0.01$. SI Figure S19b shows that the total exposure of a country E_{down}^d is still negatively correlated with its GDP per capita, $r = -0.40$, $p < 10^{-4}$.

When only the largest nodes are considered, even smaller networks are representative. In SI Fig. S18c we show DebtRank calculated on the induced subgraph of the 1% largest nodes, $R_i^{0.01,largest}$. Correlation remains large, with a Pearson correlation coefficient of $r(R_i^t, R_i^{0.01,largest}) = 0.89$ ($p \approx 0.0$) and a Spearman correlation of $\rho(R_i^t, R_i^{0.01,largest}) = 0.81$ ($p \approx 0.0$). Note that also $R_i^{0.01,largest}$ is typically larger than R_i^t, as can be seen by the coefficients of the linear fit: $R_i^{0.01,largest} = 3.2R_i^t + 2.4 \cdot 10^{-4} + \epsilon$. An examination of the scaling relation $R_i^{0.01,largest} \propto (R_i^t)^{0.90}$ (red broken line) reveals that high values of systemic risk seem to be underestimated relatively to low values. The omission of the 99% smallest nodes seems to weaken the observed effect. Also on the induced subgraph of the 1% largest firms, the main result can be reproduced with a negative correlation $r = -0.28$, $p < 0.04$, between E_{down}^d and GDP per capita (not shown).

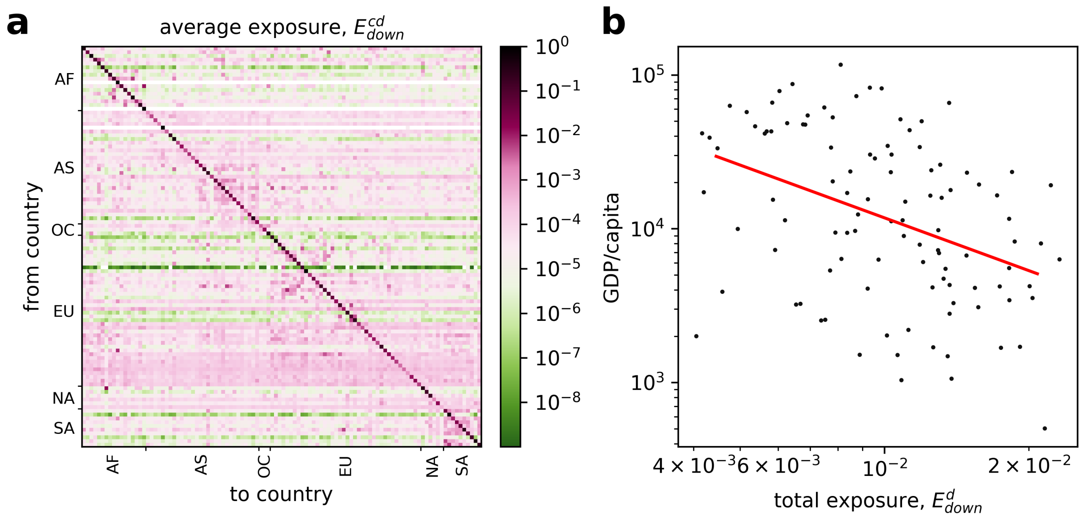

SI Fig. S19. Testing the robustness of our results using the induced subgraph of the 10% largest nodes. (a) Average exposure E^{cd}, showing the average exposure country d suffers to defaults in country c. Note that values below 10^{-10} are cropped. (b) Comparison of total exposure E^{d} and GDP per capita. The two values are negatively correlated (Pearson $r = -0.40$, $p < 10^{-4}$). The red line represents the log-log ordinary least squares regression fit to the data of form $y \sim x^{-1.14}$. The results for E^{cd} and E^{d}_{down} qualitatively reproduce the main results presented in the main text.

Removal of small links

The global supply network analyzed in this study has an average degree of $\bar{k}_{S\&P} = 5.72$, which is relatively low compared to average degrees of high-quality national supply networks derived from VAT data $\bar{k}_{VAT} \approx 60 - 100$ [12]. This discrepancy is not particularly surprising, because business intelligence databases, such as S&P Capital IQ, focus on firm's most important suppliers (and customers). In fact, to a large extent the dataset is based on so called '10-K filings' of US firms. Companies in the USA with more than \$10 million in assets and more than 2000 shareholders need to file standardized annual reports ('10-K filings') with the SEC (United States Securities and Exchange Commission) that contain, among other information, all customers which represent more than 10% of their revenue. The information of the 10-K filings is then extended using publicly available data.

There is no global supply network data available to simulate the effect of removing links based on their weights. Therefore we turn to Ecuador's national supply network, reconstructed from VAT data (see [12] for a detailed description of the dataset). The data is collected and provided by the Ecuadorian internal revenue service. It contains information on all firms and natural persons registered in the country and yearly transaction information between them reconstructed from VAT reports. Here, we use the latest available year, 2015, restrict the network to firms only and use the largest (weakly) connected component. After cleaning, the network contains $N = 86,391$ firms and $L = 3,373,835$ buyer-supplier relations. The network has an average total degree of $\bar{k}_{tot} = \bar{k}_{in} + \bar{k}_{out} = 78.1$ (note that the value in [12] differs by a factor of 2, because they define average degree in a different way, $\bar{k} = \bar{k}_{in} = \bar{k}_{out}$). We reproduce the main results of the paper by considering shock exposures between Ecuadorian provinces. First we calculate the province-level downstream exposure, E^{d}_{down}, where d represents one of the 25 provinces. Second, we obtain GDP and population data from Ecuador's National Institute of Statistics and Censuses (`https://www.ecuadorencifras.gob.ec/`). We find a negative correlation between E^{d}_{down} and $GDP/capita$ with a Pearson correlation of $r = 0.34$, however a one-sided significance test (H0: The correlation is non-negative.) is only significant at a $p < 0.1$ level. We attribute this to the small number of provinces in Ecuador. Notwithstanding, we can perform our robustness exercise and focus on firm-level DebtRank, R_i.

In our first exercise to investigate the effect of missing links, we apply a threshold of \$100,000 and drop all links that have a lower annual transaction volume. This reduces the average degree to $\bar{k}^{thr} = 5.73$, which is similar to the average degree of the global supply network ($\bar{k} = 5.72$, see SI Tab. S4). Supplementary Figure S20a plots DebtRank on the full Ecuadorian supply network, R^{t}_{i}, against DebtRank on the thresholded network, R^{thr}_{i}. The results on the thresholded and the full network are highly correlated, with a Pearson coefficient of $r(R^{t}_{i}, R^{thr}_{i}) = 0.82$ ($p \approx 0.0$) and a Spearman correlation of $\rho(R^{t}_{i}, R^{thr}_{i}) = 0.65$ ($p \approx 0.0$). Note that R^{thr}_{i} is typically larger than R^{t}_{i}, as can be seen by the coefficient of the linear fit: $R^{thr}_{i} = 1.9 R^{t}_{i} + 8.6 \cdot 10^{-5} + \epsilon$. An examination of the scaling relation $R^{thr}_{i} \propto (R^{t}_{i})^{1.16}$ (red broken line) reveals that high values of systemic risk seem to be overestimated relatively to low values. The removal of low-weight links might cause an overestimation of heterogeneity in exposure to supply chain shocks.

In a second exercise, we filter the Ecuadorian production network by removing all links that represent less than

10% of the supplier's sales, thereby emulating a reconstructed network that is solely based on 10-K filings. The average degree drops to $\bar{k}^{10K} = 3.66$, suggesting that S&P's Capital IQ database contains additional information beyond the contents of public 10-K filings. In SI Fig. S20b we compare DebtRank on the full Ecuadorian supply network, R_i^t, and DebtRank the filtered network, R_i^{10K}. The indices on the 'true' and filtered networks are moderately correlated, with a Pearson coefficient of $r(R_i^t, R_i^{10K}) = 0.58$ ($p \approx 0.0$) and a Spearman correlation of $\rho(R_i^t, R_i^{thr}) = 0.45$ ($p \approx 0.0$). Note that for large R_i, R_i^{10K} is typically smaller than R_i^t, as can be seen by the coefficient of the linear fit: $R_i^{10K} = 0.3R_i^t + 1.1 \cdot 10^{-5} + \epsilon$. An examination of the scaling relation $R_i^{10K} \propto (R_i^t)^{1.33}$ (red broken line) reveals that high values of systemic risk seem to be overestimated relatively to low values. The removal of low-weight links might cause an overestimation of heterogeneity in exposure to supply chain shocks.

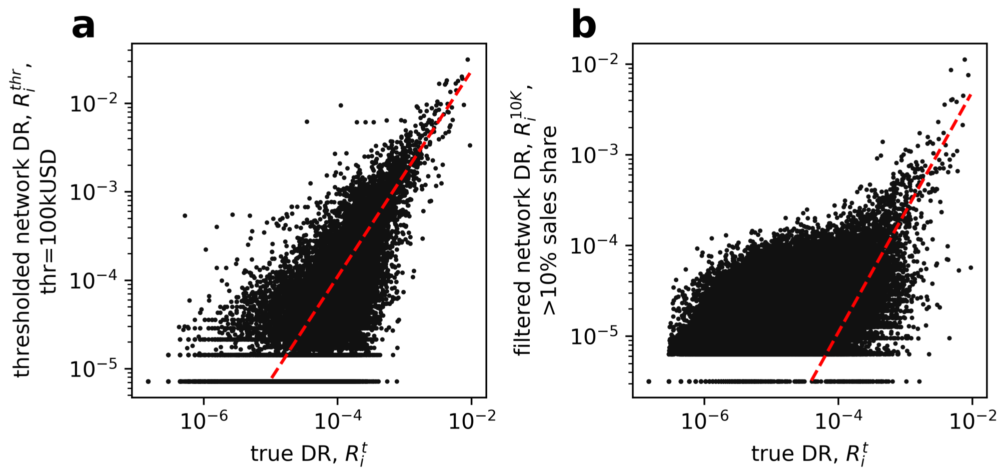

SI Fig. S20. Effect of missing links in the Ecuadorian supply network. (a) DebtRank calculated on the full Ecuadorian supply network, R_i^t, and on the same network considering only links above \$100,000 annual transaction volume, R_i^{thr}. The red broken line shows a best fit $R_i^{0.01,largest} \propto (R_i^t)^{1.16}$. (b) DebtRank calculated on the full Ecuadorian supply network, R_i^t, and on the same network considering only links that represent more than 10% of the supplier's sales share, R_i^{10K}. The red broken line shows a best fit $R_i^{0.01,largest} \propto (R_i^t)^{1.33}$. For both sampling strategies, DebtRank calculated for the sample is highly representative of a node's DebtRank on the full network.

Removal of link weights

Finally, we investigate the effect of omitting link weights by comparing DebtRank calculated using link weights, R_i^w, with DebtRank calculated on the unweighted network, R_i^u. For the weighted network, the link weights in eq. (3) take the form $w_{ji} = W_{ji}/s_i^{in}$, where W_{ji} represents the annual transaction volume.

Supplementary Figure S21a shows R_i^w and R_i^u calculated on the full Ecuadorian supply network plotted against each other. We find that the indices are highly correlated, with a Pearson coefficient of $r(R_i^w, R_i^u) = 0.69$ ($p \approx 0.0$) and a Spearman correlation of $\rho(R_i^w, R_i^u) = 0.88$ ($p \approx 0.0$). Note that R_i^w is typically smaller than R_i^u for small R_i and greater for large R_i, respectively, as can be seen by the coefficients of the linear fit: $R_i^u = 0.19R_i^w + 3.9 \cdot 10^{-5} + \epsilon$. An examination of the scaling relation $R_i^w \propto (R_i^u)^{0.58}$ (red broken line) reveals that high values of systemic risk seem to be underestimated relatively to low values. The omission of link weights seems to strongly weaken the observed effect.

As mentioned before, the global supply network used in our study is considerably less dense than the (unfiltered) Ecuadorian supply network. To investigate the interplay of omitting links and weights, we drop links with annual transaction volume below \$100,000 (same threshold as in SI Fig. S20a) and calculate Debtrank with and without considering link weights, $R_i^{u,thr}$ and $R_i^{w,thr}$, respectively. In SI Fig. S21 we plot $R_i^{u,thr}$ against $R_i^{w,thr}$. The correlation increases compared to the unfiltered network, with a Pearson coefficient of $r(R_i^{w,thr}, R_i^{u,thr}) = 0.84$ ($p \approx 0.0$) and a Spearman correlation of $\rho(R_i^{w,thr}, R_i^{u,thr}) = 0.89$ ($p \approx 0.0$). Note that, again, $R_i^{w,thr}$ is typically smaller than $R_i^{u,thr}$ for small R_i and greater for large R_i, respectively, as can be seen by the coefficients of the linear fit: $R_i^{u,thr} = 0.43R_i^w + 8.1 \cdot 10^{-5} + \epsilon$. An examination of the scaling relation $R_i^{w,thr} \propto (R_i^{u,thr})^{0.73}$ (red broken line) reveals that high values of systemic risk seem to be underestimated relatively to low values. The omission of link weights seems to dominate over the effect of the removal of low-weight links and results in an overall underestimation of the observed heterogeneity in shock exposures.

Last, but not least, we can revisit the question how representative a firm's degree is for its sales, especially in the context of missing links. In SI Fig. S22 we plot every firm's sales S_i against its degree on the full, unfiltered

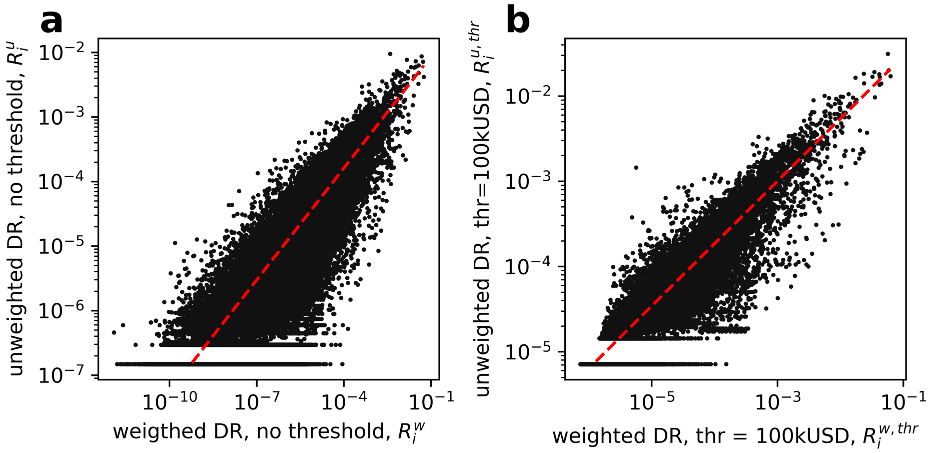

SI Fig. S21. Effect of omitting links weights in the Ecuadorian supply network. (a) DebtRank calculated on the weighted Ecuadorian supply network, R_i^w, and on the same network considering all links equal weight, R_i^u. The red broken line shows a best fit $R_i^{0.1,unif} \propto (R_i^t)^{0.58}$. (b) DebtRank calculated on the Ecuadorian supply network considering only links above \$100,000, with, $R_i^{w,thr}$, and without link weights, $R_i^{u,thr}$, respectively. The red broken line shows a best fit $R_i^{0.1,unif} \propto (R_i^t)^{0.73}$. For both the full and the thresholded network, unweighted DebtRank is highly representative of weighted DebtRank.

network (grey), k_i^0, and on the network where we omit all links below an annual transaction volume of \$100,000 (red), k_i^{thr}. Without a link weight threshold, sales and degree are moderately correlated, with a Pearson coefficient of $r(k^0, S) = 0.40$ ($p \approx 0.0$) and a Spearman correlation of $\rho(k^0, S) = 0.65$ ($p \approx 0.0$). After thresholding, the correlation increases substantially to $r(k^{thr}, S) = 0.72$ ($p \approx 0.0$) and $\rho(k^0, S) = 0.83$ ($p \approx 0.0$). It appears that removing low-weight links removes noise and increases the correlation between sales and degree. Note, that for the unfiltered network a firm's sales, S_i, corresponds to its outstrength, s_i^{out}.

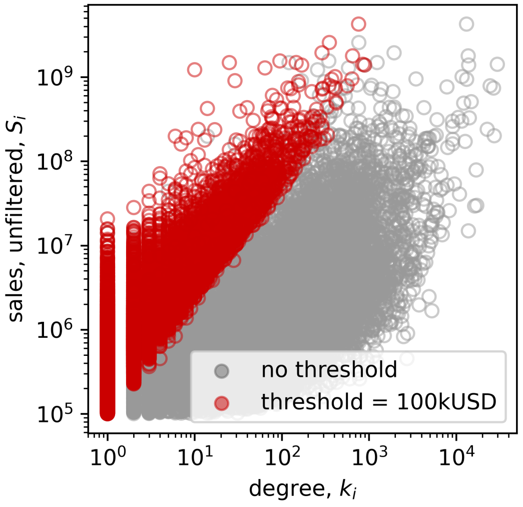

SI Fig. S22. Representativeness of a firm's degree for its sales. Degree of firms in the full Ecuadorian supply network (grey), k_i^0, compared to firms' degrees on a network where only links with an annual transaction volume greater than \$100,000 are considered, k_i^{thr}, compared to the nodes' sales, S_i. The correlation between degree and sales increases significantly after thresholding.

SI Text 13: Relationship between aggregate firms degree and macroeconomic variables

Our dataset lacks information on the traded volumes (i.e. link weights) and firm level information such as revenue. We assume equal weights on all links ($A_{ij} \in \{0,1\}$) and verify that this approximation is justified on the aggregate level by comparing it to macroeconomic variables. We calculate the total degree k^c, total export links L_e^c, and total import links L_i^c of firms in a country. The total degree for a country c, is measured as the total number of links of all firms in country c. L_e^c represents the total number of outgoing links of all firms in c to firms in all other countries. Similarly, L_i^c represents the total number of incoming links of all firms in the c from firms in all other countries. SI Figure S23 shows that these quantities are strongly correlated with GDP, total exports and total imports with the Pearson correlation coefficients $r = 0.87$, $p < 10^{-15}$, $r = 0.77$, $p < 10^{-15}$ and $r = 0.86$, $p < 10^{-15}$ respectively.

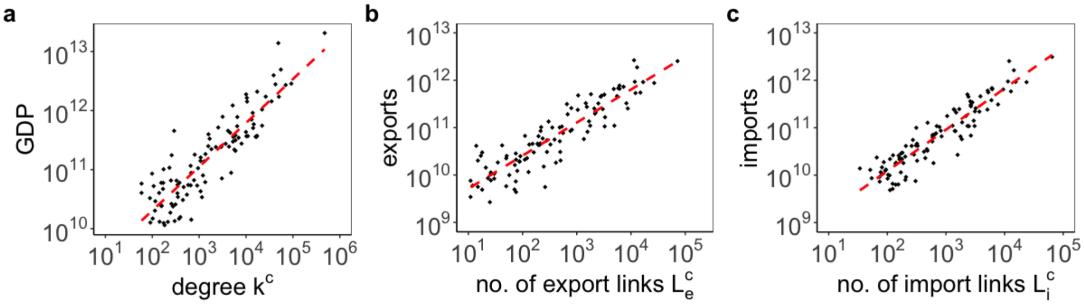

SI Fig. S23. Comparison of country-aggregated degree and macroeconomic variables. (a) Total degree k^c plotted against GDP. The red dotted line represents $GDP \sim (k^c)^{0.74}$ (b) Number of links out of a country L_e^c compared to its export volume. The red dotted line represents Exports $\sim (L_e^c)^{0.69}$ (c) Number of links into a country L_i^c compared to its import volume. The red dotted line represents Imports $\sim (L_i^c)^{0.87}$. All in current USD. All investigated quantities correlate strongly and suggest a relationship between size and representation in the supply network.

SI Text 14: Results for states in the USA

Here we study downstream shock propagation in the national supply network of the USA, aggregated to the 50 states of the USA. In SI Fig. S24 we plot the exposure matrix E^{cd}_{down} between US states, sorted according to the regional classification by the US census bureau. Here, similarly to the exposures in the global supply network, the strongest exposures are along the diagonal –within the respective states–. However, we do not observe a pronounced block structure, indicating that exposures are not stronger within geographic regions. This can be explained by less pronounced regional differences, a shared cultural history and a lack of trade barriers within the US. The more pronounced structure is that large, rich states create a lot of exposure to the rest of the country, visible as bright and magenta horizontal lines.

SI Figure S25 (a) shows that the states with lower GDP per capita receive more total exposure E^d_{down} than states with higher GDP per capita. Albeit the correlation being insignificant ($r = -0.16, p = 0.28$), the data shows a weak negative relationship between GDP per capita and E^d, as indicated by the red trend line in SI Fig. S25 (a). We investigate the inequality in total exposure E^d_{down} and GDP for US states with the Lorentz curves in SI Fig. S25 (b). The Gini coefficients are found to be $= 0.54$ and 0.11 for total exposure and GDP, respectively. This indicates that, albeit less than for the global supply network, total downstream exposure is more concentrated than GDP among states in USA. We conclude that, despite lower inter-state heterogeneity we find visually similar results to the global supply network.

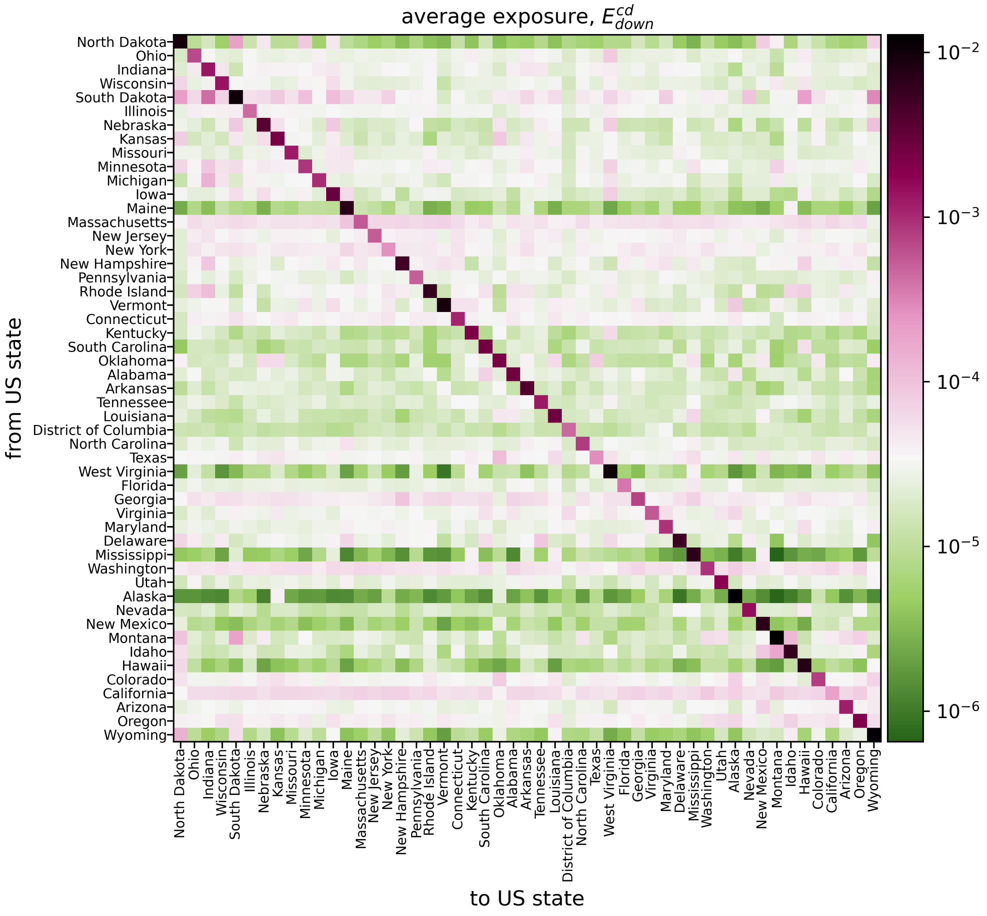

SI Fig. S24. Exposures between US states in the national supply network, aggregated to US state level. The expected fraction of the economy affected in US state d, subsequent to an arbitrary firm default in state c, E^{cd}_{down}, plotted on a logarithmic scale. The countries are sorted by US region (as defined by the US census bureau) in the following order: Midwest, Northeast, South, West. The values are highest along the diagonal, but in contrast to the to the global supply network the there are no regional blocks of high exposure. This can be explained by the history, as well as the physical and economic geography of the US.

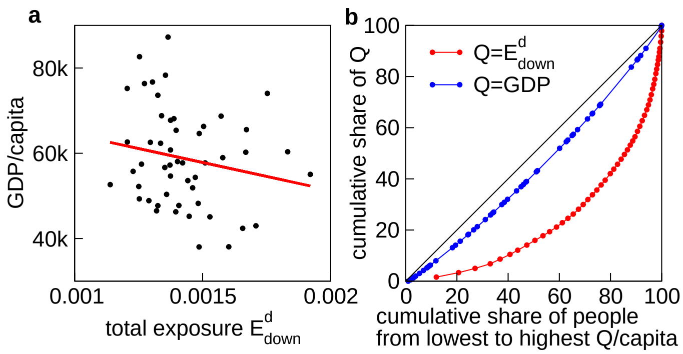

SI Fig. S25. (a) Variation of total exposure with state GDP per capita of USA. The red line represents the best linear fit to the data of form $y = 0.08 - 13.1x$. (b) Lorenz curves for total exposure and GDP of all the states of USA. The Gini coefficients = 0.54 and 0.11 for total exposure E^d_{down} and GDP respectively. GDP data are obtained from Bureau of Economic Analysis `https://www.bea.gov`, and population data are obtained from U.S. Census Bureau, Population Division `https://www.census.gov`.

SI Text 15: Network indicators

Here we provide a short collection of network summary statistics of the global supply network used in this paper. Table S4 shows the values for the number of nodes and links, the density of the network, and the averages for the degree, the in- and outdegree, the nearest-neighbor degree and the local clustering coefficient.

In Fig. S26a we show the counter-cumulative distribution of the degree. The degree distribution is fat-tailed. Figure S26b shows binned averages of the nearest-neighbor degrees for logarithmic degree-bins. The network is weakly disassortative. Figure S26c shows binned averages of the nearest-neighbor degrees for logarithmic degree-bins. The (average) local clustering coefficient scales negatively with the degree.

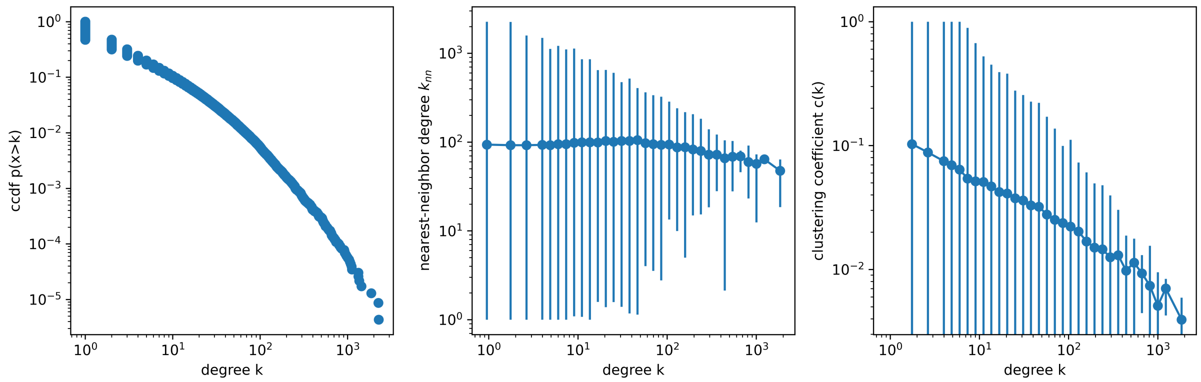

SI Fig. S26. Supply network topology. (a) Counter-cumulative degree distribution. (b) Averages and minimum-maximum spread of nearest-neighbor degrees and (c) clustering coefficients as a function of degree (log bins).

TABLE S4. Network summary statistics.

description	symbol	value
number of nodes	N	230970
number of links	L	660701
density	ρ	0.000025
average degree	$\langle k \rangle$	5.72
average indegree	$\langle k_{in} \rangle$	2.86
average outdegree	$\langle k_{out} \rangle$	2.86
average nearest-neighbor degree	$\langle k_{NN} \rangle$	11.98
average clustering coefficient	$\langle c_i \rangle$	0.02

[1] Y. Fujiwara, M. Terai, Y. Fujita, and W. Souma, RIETI Discussion Paper Series **16-E-046** (2016).

[2] J.-N. Barrot and J. Sauvagnat, The Quarterly Journal of Economics **131**, 1543 (2016).

[3] V. M. Carvalho, M. Nirei, Y. U. Saito, and A. Tahbaz-Salehi, The Quarterly Journal of Economics **136**, 1255 (2021).

[4] C. E. Boehm, A. Flaaen, and N. Pandalai-Nayar, Review of Economics and Statistics **101**, 60 (2019).

[5] S. Poledna, J. L. Molina-Borboa, S. Martínez-Jaramillo, M. Van Der Leij, and S. Thurner, Journal of Financial Stability **20**, 70 (2015).

[6] J. E. Anderson, Annual Review of Economics **3**, 133 (2011), https://doi.org/10.1146/annurev-economics-111809-125114.

[7] A.-L. Wölwer, M. Breßlein, and J. P. Burgard, Austrian Journal of Statistics **47**, 16 (2018).

[8] H. Van den Berg and J. J. Lewer, *International Trade and Economic Growth* (Routledge, 2015).

[9] M. Ramzan, B. Sheng, M. Shahbaz, J. Song, and Z. Jiao, The Journal of International Trade & Economic Development **28**, 960 (2019), https://doi.org/10.1080/09638199.2019.1616805.

[10] E. Borensztein, J. De Gregorio, and J.-W. Lee, Journal of International Economics **45**, 115 (1998).

[11] A. Pichler, C. Diem, A. Brintrup, F. Lafond, G. Magerman, G. Buiten, T. Y. Choi, V. M. Carvalho, J. D. Farmer, and S. Thurner, Science **382**, 270 (2023), https://www.science.org/doi/pdf/10.1126/science.adi7521.

[12] A. Bacilieri, A. Borsos, P. Astudillo-Estevez, and F. Lafond, *Firm-level production networks: what do we (really) know*, Tech. Rep. (mimeo, University of Oxford, 2022).
