# Supplementary material for: Inequality in economic shock exposures across the global firm-level supply network
Source: Nat Commun. 2024 Apr 18;15:3348. doi: 10.1038/s41467-024-46126-w (PMC11026497; doi:10.1038/s41467-024-46126-w)
Supplement: Supplementary file 4 — Source Data [file 41467_2024_46126_MOESM4_ESM.zip › Manuscript source data.zip!/manuscript_format_changes/Figures/fig2/fig3-04a-horizontal4colors_expected.pdf]

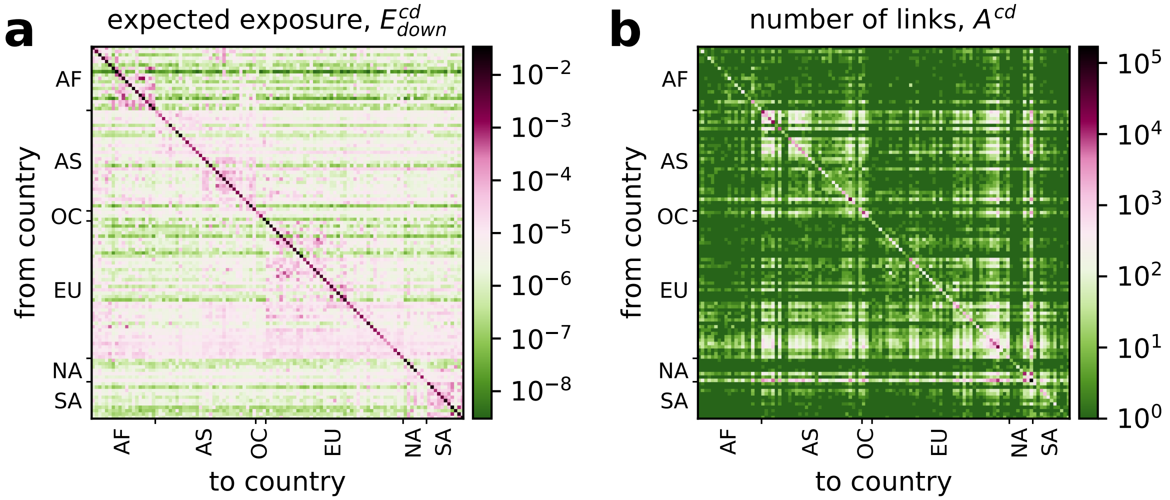

a
expected exposure, E^{cd}_{down}
AF
AS
OC
EU
NA
SA
from country
to country
10^{-2}
10^{-3}
10^{-4}
10^{-5}
10^{-6}
10^{-7}
10^{-8}
b
number of links, A^{cd}
10^{5}
10^{4}
10^{3}
10^{2}
10^{1}
10^{0}
