# Supplementary material for: Inequality in economic shock exposures across the global firm-level supply network
Source: Nat Commun. 2024 Apr 18;15:3348. doi: 10.1038/s41467-024-46126-w (PMC11026497; doi:10.1038/s41467-024-46126-w)
Supplement: Supplementary file 4 — Source Data [file 41467_2024_46126_MOESM4_ESM.zip › Manuscript source data.zip!/manuscript_format_changes/Figures/supplementary/S4-av_gdp_growth_rate_vs_exposure.pdf]

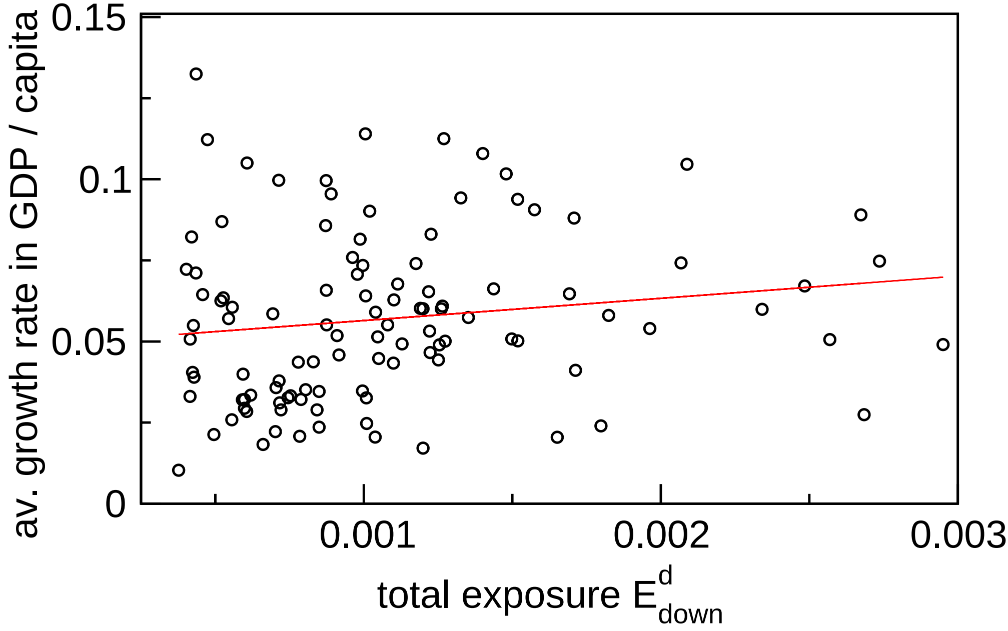

av. growth rate in GDP / capita
0.15
0.1
0.05
0
0.001
0.002
0.003
total exposure E^{d}_{down}
