# Supplementary material for: Inequality in economic shock exposures across the global firm-level supply network
Source: Nat Commun. 2024 Apr 18;15:3348. doi: 10.1038/s41467-024-46126-w (PMC11026497; doi:10.1038/s41467-024-46126-w)
Supplement: Supplementary file 4 — Source Data [file 41467_2024_46126_MOESM4_ESM.zip › Manuscript source data.zip!/manuscript_format_changes/Figures/supplementary/S8-Gini_capita_upstream_v2.pdf]

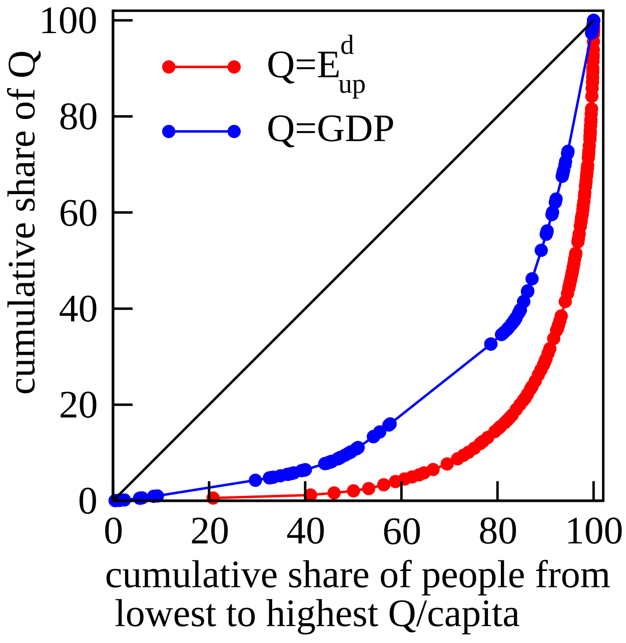

$Q=E_{up}^{d}$
Q=GDP
cumulative share of Q
cumulative share of people from lowest to highest Q/capita
0
20
40
60
80
100
