# Supplementary material for: Inequality in economic shock exposures across the global firm-level supply network
Source: Nat Commun. 2024 Apr 18;15:3348. doi: 10.1038/s41467-024-46126-w (PMC11026497; doi:10.1038/s41467-024-46126-w)
Supplement: Supplementary file 4 — Source Data [file 41467_2024_46126_MOESM4_ESM.zip › Manuscript source data.zip!/manuscript_format_changes/Figures/supplementary/S11-Gini_capita_USA_v2.pdf]

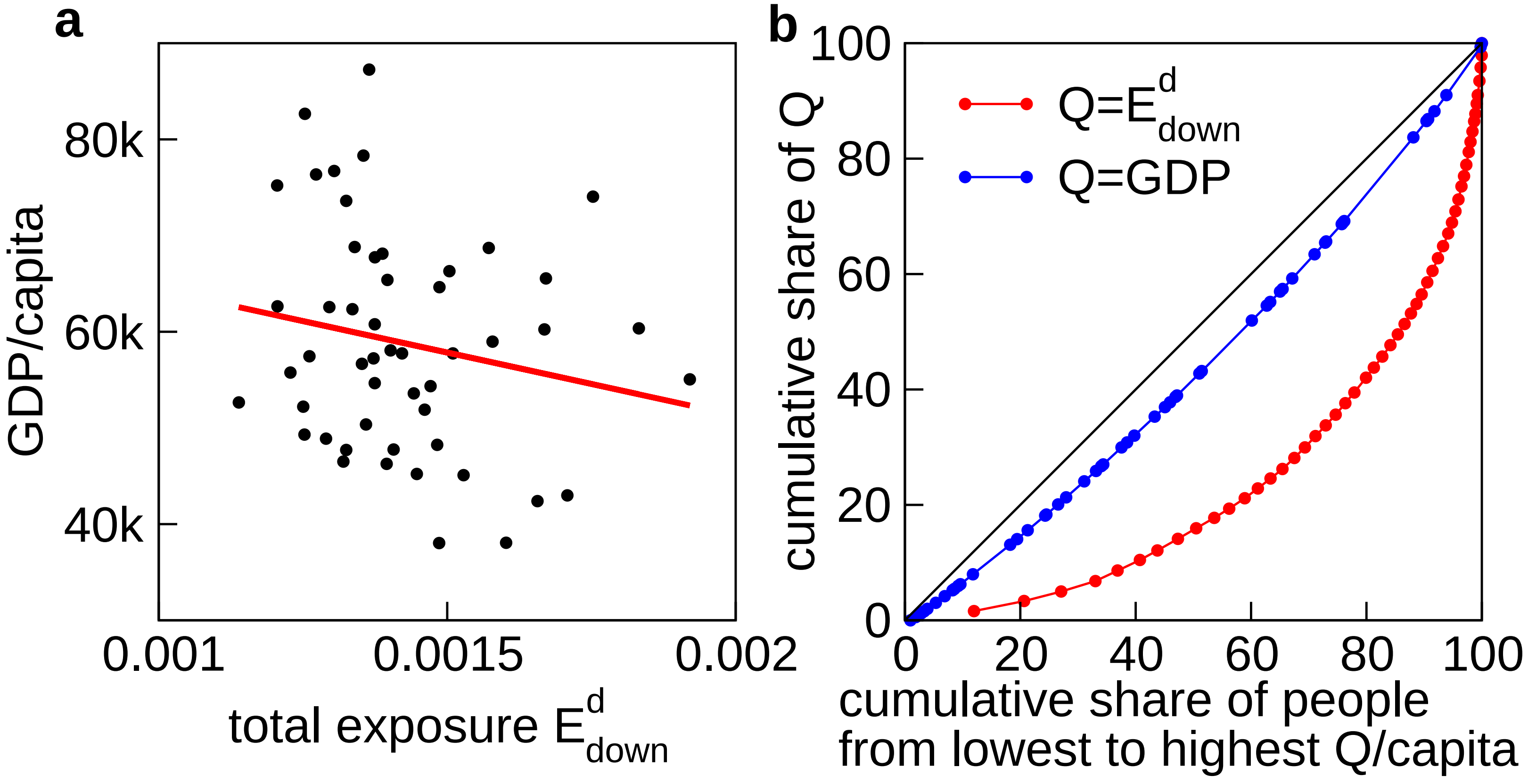
a
GDP/capita
80k
60k
40k
0.001
0.0015
0.002
total exposure E^{d}_{down}
b
cumulative share of Q
100
80
60
40
20
0
$Q=E^{d}_{down}$
Q=GDP
0
20
40
60
80
100
cumulative share of people
from lowest to highest Q/capita
