# Supplementary material for: Inequality in economic shock exposures across the global firm-level supply network
Source: Nat Commun. 2024 Apr 18;15:3348. doi: 10.1038/s41467-024-46126-w (PMC11026497; doi:10.1038/s41467-024-46126-w)
Supplement: Supplementary file 4 — Source Data [file 41467_2024_46126_MOESM4_ESM.zip › Manuscript source data.zip!/manuscript_format_changes/Figures/supplementary/S6-SI_upstream_exposurematrix_large-01.pdf]

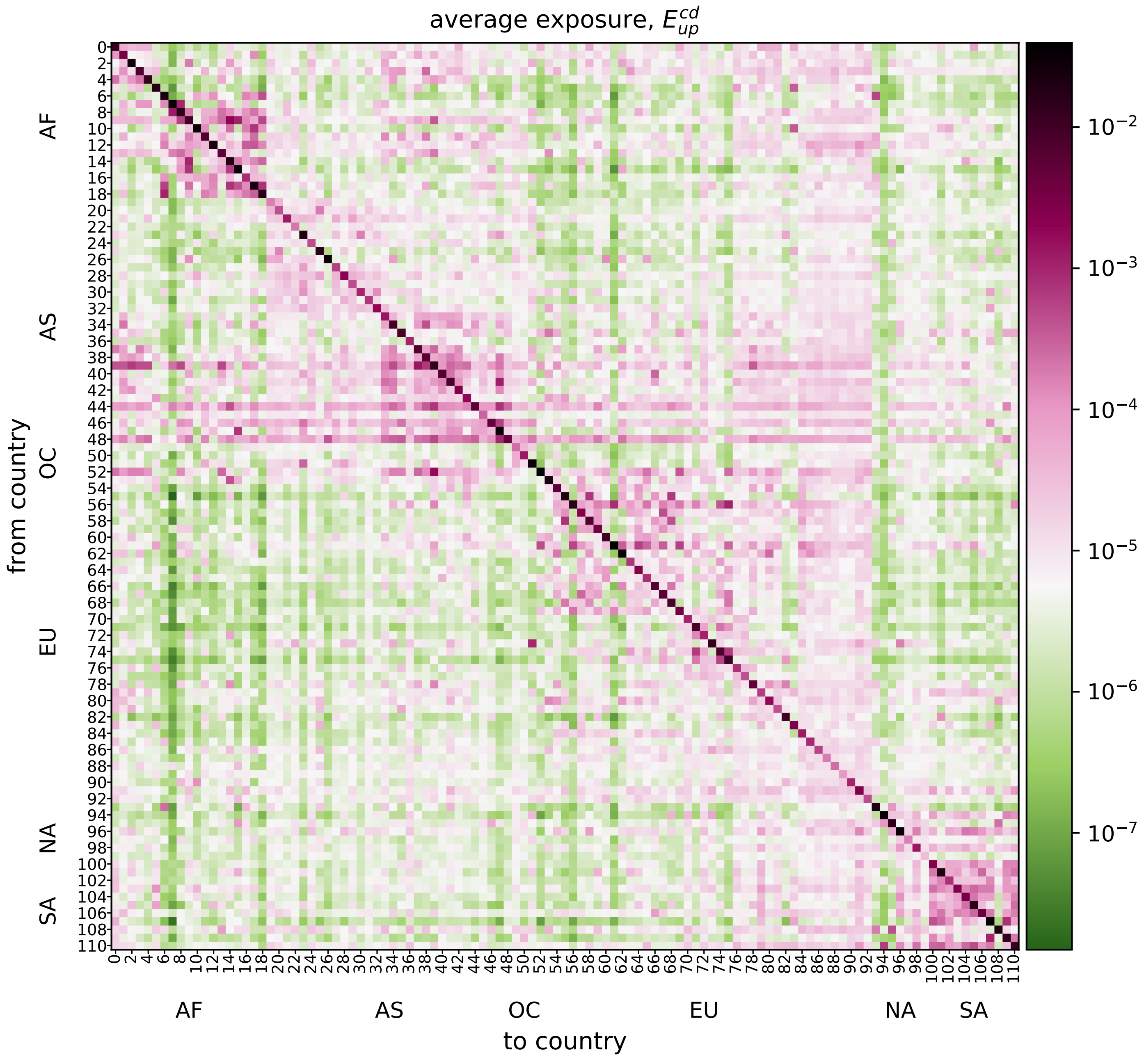

average exposure, E_{up}^{cd}
from country
to country
AF
AS
OC
EU
NA
SA
10^{-2}
10^{-3}
10^{-4}
10^{-5}
10^{-6}
10^{-7}
