# Supplementary material for: Inequality in economic shock exposures across the global firm-level supply network
Source: Nat Commun. 2024 Apr 18;15:3348. doi: 10.1038/s41467-024-46126-w (PMC11026497; doi:10.1038/s41467-024-46126-w)
Supplement: Supplementary file 4 — Source Data [file 41467_2024_46126_MOESM4_ESM.zip › Manuscript source data.zip!/manuscript_format_changes/Figures/supplementary/S10-SI_US_exposurematrix_large-01.pdf]

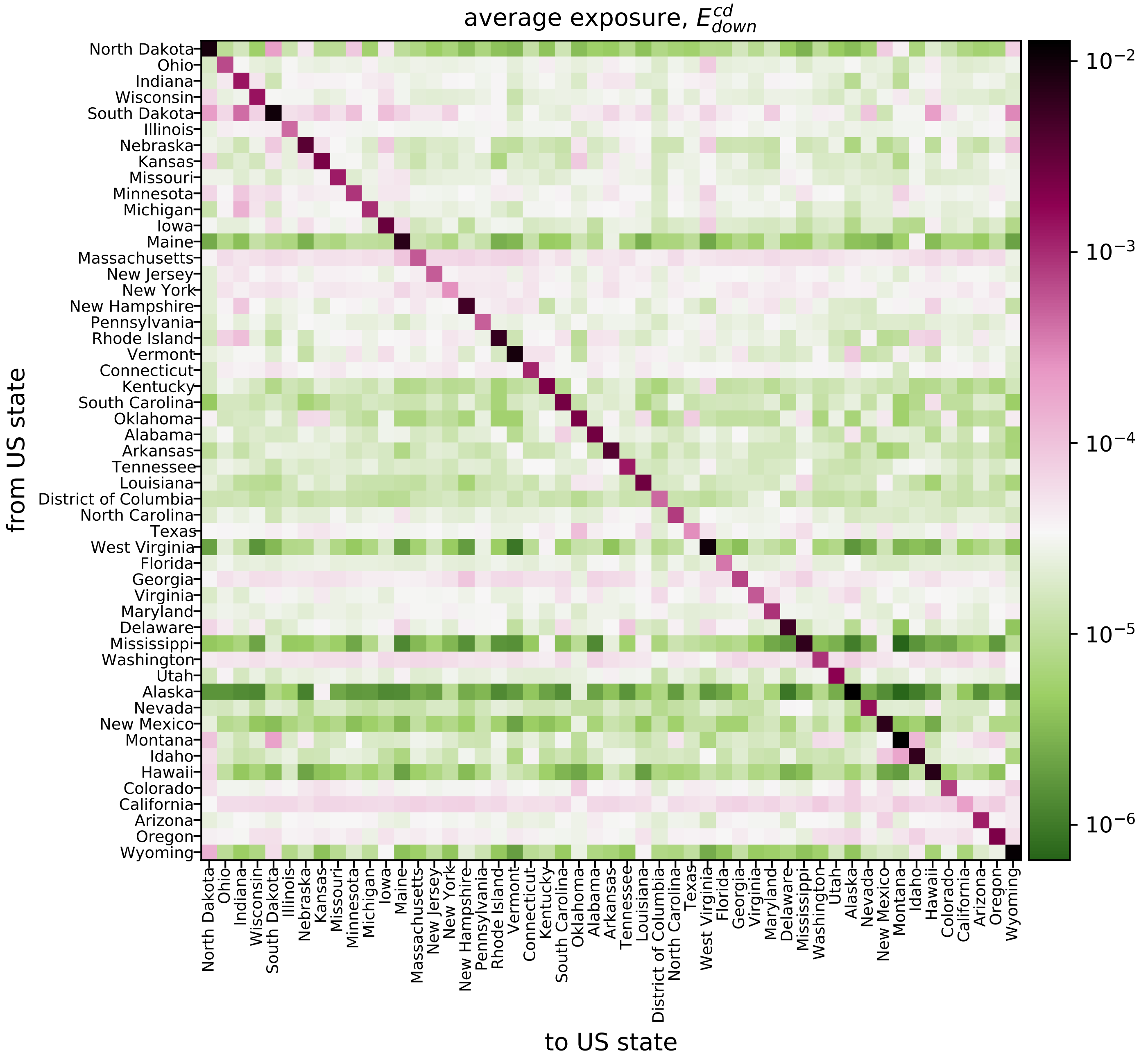
average exposure, E^{cd}_{down}
from US state
to US state
North Dakota
Ohio
Indiana
Wisconsin
South Dakota
Illinois
Nebraska
Kansas
Missouri
Minnesota
Michigan
Iowa
Maine
Massachusetts
New Jersey
New York
New Hampshire
Pennsylvania
Rhode Island
Vermont
Connecticut
Kentucky
South Carolina
Oklahoma
Alabama
Arkansas
Tennessee
Louisiana
District of Columbia
North Carolina
Texas
West Virginia
Florida
Georgia
Virginia
Maryland
Delaware
Mississippi
Washington
Utah
Alaska
Nevada
New Mexico
Montana
Idaho
Hawaii
Colorado
California
Arizona
Oregon
Wyoming
10^{-2}
10^{-3}
10^{-4}
10^{-5}
10^{-6}
