# Supplementary material for: Inequality in economic shock exposures across the global firm-level supply network
Source: Nat Commun. 2024 Apr 18;15:3348. doi: 10.1038/s41467-024-46126-w (PMC11026497; doi:10.1038/s41467-024-46126-w)
Supplement: Supplementary file 4 — Source Data [file 41467_2024_46126_MOESM4_ESM.zip › Manuscript source data.zip!/manuscript_format_changes/Figures/supplementary/S7-SI-01-upstream_incomegroups_scatter.pdf]

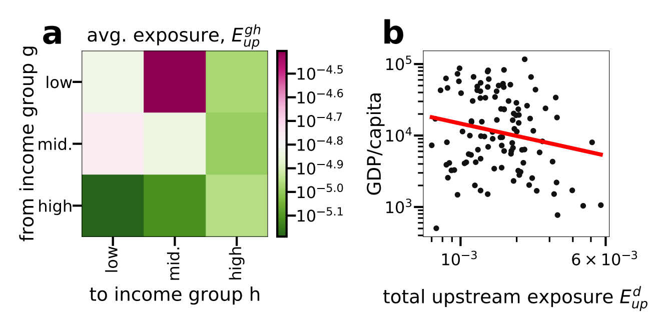
a
avg. exposure, E_{up}^{gh}
from income group g
low
mid.
high
to income group h
low
mid.
high
$10^{-4.5}$
$10^{-4.6}$
$10^{-4.7}$
$10^{-4.8}$
$10^{-4.9}$
$10^{-5.0}$
$10^{-5.1}$
b
GDP/capita
10^5
10^4
10^3
10^{-3}
6×10^{-3}
total upstream exposure E_{up}^{d}
