# Supplementary material for: Inequality in economic shock exposures across the global firm-level supply network
Source: Nat Commun. 2024 Apr 18;15:3348. doi: 10.1038/s41467-024-46126-w (PMC11026497; doi:10.1038/s41467-024-46126-w)
Supplement: Supplementary file 4 — Source Data [file 41467_2024_46126_MOESM4_ESM.zip › Manuscript source data.zip!/manuscript_format_changes/Figures/fig1/fig1-03-PiYG.pdf]

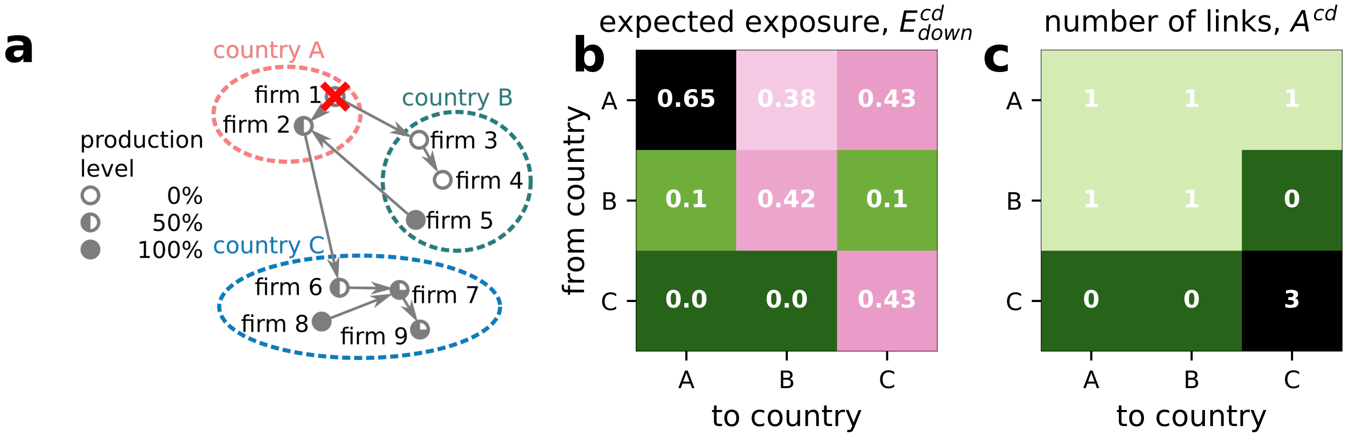

a
country A
firm 1
firm 2
country B
firm 3
firm 4
firm 5
production level
0%
50%
100%
country C
firm 6
firm 7
firm 8
firm 9
b
expected exposure, E^{cd}_{down}
from country
to country
A B C
0.65 0.38 0.43
0.1 0.42 0.1
0.0 0.0 0.43
c
number of links, A^{cd}
1 1 1
1 1 0
0 0 3
