# Supplementary material for: Inequality in economic shock exposures across the global firm-level supply network
Source: Nat Commun. 2024 Apr 18;15:3348. doi: 10.1038/s41467-024-46126-w (PMC11026497; doi:10.1038/s41467-024-46126-w)
Supplement: Supplementary file 4 — Source Data [file 41467_2024_46126_MOESM4_ESM.zip › Manuscript source data.zip!/manuscript_format_changes/Figures/fig3/fig4-02-2panels_incomegroups_scatter.pdf]

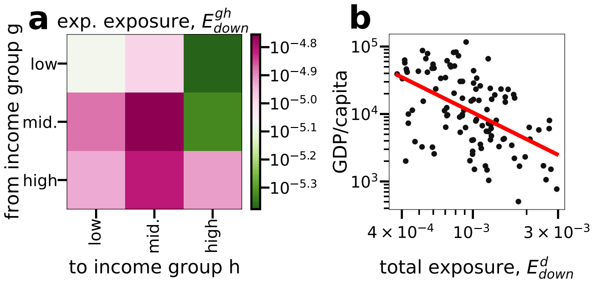
a
exp. exposure, E^{gh}_{down}
from income group g
low
mid.
high
to income group h
low
mid.
high
$10^{-4.8}$
$10^{-4.9}$
$10^{-5.0}$
$10^{-5.1}$
$10^{-5.2}$
$10^{-5.3}$
b
GDP/capita
10^5
10^4
10^3
4×10^{-4}
10^{-3}
3×10^{-3}
total exposure, E^{d}_{down}
